# *In vitro* evaluation of the immunogenic potential of gramicidin S and its photocontrolled analogues†

**DOI:** 10.1039/d5md00075k

**Published:** 2025-04-10

**Authors:** Kateryna Horbatok, Iryna Semchuk, Oleksandr Horbach, Natalia Khranovska, Viktoriia Kosach, Petro Borysko, Serhii Koniev, Anne S. Ulrich, Sergii Afonin, Igor V. Komarov

**Affiliations:** a Taras Shevchenko National University of Kyiv Volodymyrska street 60 01601 Kyiv Ukraine igor.komarov@knu.ua; b Enamine Ltd. Winston Churchill street 78 02094 Kyiv Ukraine; c Nonprofit organization “National Cancer Institute” Yulii Zdanovskoi street 33/43 03022 Kyiv Ukraine; d V. P. Kukhar Institute of Bioorganic Chemistry and Petrochemistry Akademician Kukhar street 1 02094 Kyiv Ukraine; e Karlsruhe Institute of Technology POB 3640 76021 Karlsruhe Germany sergiy.afonin@kit.edu; f Lumobiotics Auerstraße 2 76227 Karlsruhe Germany

## Abstract

Three hallmarks of ICD (immunogenic cell death), release of adenosine triphosphate (ATP), release of high mobility group box 1 protein, and calreticulin exposure on the cell surface, were studied upon treatment of mammalian cells with small cyclic peptides, namely, the natural antibiotic gramicidin S (GS) and two photocontrolled GS analogues (LMB002 and LMB033). The analogues contained a photoisomerizable diarylethene fragment, and they exhibited different bioactivities in their “open” and “closed” photoisomeric forms. The data (obtained from cell cultures and spheroids) were collected in a concentration-dependent manner to assess cytotoxicity. Results showed that treatment with all peptides induced ICD at sub-IC_50_ and higher concentrations, indicating that GS and its derivatives have promising immunogenic potential. The “open” photoisomers of the photoswitchable GS analogues generated using visible light were as efficient as ICD inducers and the parent GS, while the UV-generated “closed” photoforms induced ICD only at higher concentrations. Herein, the cell specificity and time dependency of the observed effects are presented.

## Abstract 2

The key markers of immunogenic cell death were detected in mammalian cell cultures upon co-incubation with gramicidin S and its photoswitchable analogues at the sub-membranolytic concentrations, suggesting an immunotherapeutic potential for peptides.

## Graphical abstract

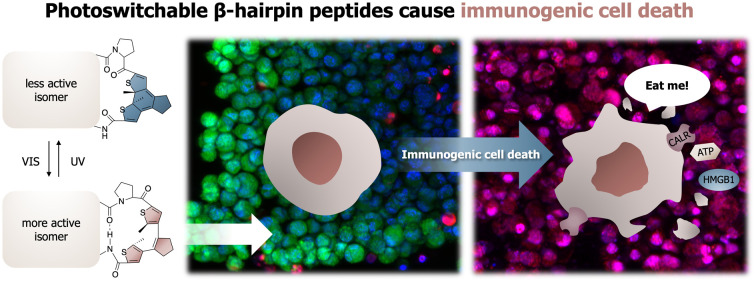

## Introduction

Cancer chemotherapy has long been considered to be solely immunosuppressive by most scientists,^1^ although indirect evidences exist for the active contribution of immune effectors to its positive outcome.^2^ These evidences started to accumulate since the 18th century; a prominent historical example is the mixed bacterial vaccine Coley's toxin that has been used since the end of the 19th century as a nonspecific immunotherapeutic to treat inoperable cancer.^3^ However, this cancer treatment approach has been suspended owing to the success of chemotherapy, surgery and radiotherapy.^4^ Only since the turn of the 21st century, the interest to immunotherapy reawakened, as a growing number of studies demonstrated that many cytotoxic agents may elicit powerful antitumor immune responses.^2,3^ It was found that the efficacy of “conventional” cytotoxic anticancer drugs, such as anthracyclines, bleomycin, and oxaliplatin, benefits from considerable immunostimulation as they impact the whole-body physiology, and accordingly, a variety of on-target effects were united under the concept of “Immunogenic Cell Death” (ICD).^4^ Pioneering studies triggered extensive search on efficient inducers of immunostimulation of this kind, and opened exciting possibilities for recruiting the immune system as a powerful ally in cancer treatment, either alone or in combination with emerging tools of cancer immunotherapy.^5–9^ It was demonstrated that ICD inducers include not only chemotherapeutic agents but also other cancer treatment modalities, some of which were used in clinical practice for a long time, for example, treatment with oncolytic viruses,^10^ radiotherapy^11^ and photodynamic therapy.^12^

ICD is now regarded crucial for the *in vivo* immunostimulation, and molecular mechanisms leading to it are being thoroughly investigated. The concept of ICD has developed over the years, and there is now a consensus among scientists for its definition, detection, and interpretation.^13^ According to this consensus, ICD is “a form of regulated cell death that is sufficient to activate an adaptive immune response in immunocompetent syngeneic hosts”.^14^ ICD is not a mere activation of the stress-induced innate immune response: to awaken a robust adaptive response, the dying tumor cells should provide a sufficient level of antigenicity and adjuvanticity.

Antigenicity in the context of ICD refers to the ability of a cell to generate and present the antigens that can be recognized by naïve T cell clones. Major sources of antigenicity of cancer cells include tumor-associated antigens^15^ and tumor neoantigens.^16^ However, cancer cells have evolved to evade or damp the antigen-presentation by numerous ways;^17^ therefore, additional activation is needed to elicit adequate immune responses. This additional activation is embodied by adjuvanticity provided by the molecules which dying cells release or expose on their surface. These molecules are called danger-associated molecular patterns (DAMPs); they are necessary for the recruitment and maturation of antigen-presenting cells. ATP, nucleic acids, non-histone DNA-binding protein high mobility group box 1 (HMGB1), annexin A1, certain cytokines (type I interferon, CCL2, CXCL1, and CXCL10), endoplasmic reticulum chaperones (*e.g.* calreticulin), heat shock proteins (HSP70 and HSP90), reactive oxygen species (ROS), cytosolic F-actin, mitochondrial DNA, and cardiolipin are proven examples of the DAMPs.^13^ Importantly, in the absence of adjuvanticity, the death of tumor cells, even being accompanied by sufficient antigenicity, may not lead to a sustainable anticancer effect.^18^

Among the compounds which were experimentally confirmed to induce ICD, lytic peptides possess a prominent place.^19^ Some of the lytic peptides now undergo preclinical or clinical evaluation as anticancer drugs capable of stimulating immune response.^20,21^ They demonstrated a synergy in combination with the immune checkpoint inhibitors and are considered promising in other strategies of cancer treatment. These peptides are either natural cationic host-defense peptides or their synthetic analogues. They act mainly through receptor-independent, membrane-destabilizing mechanisms.

Although lytic peptides have already demonstrated promising therapeutic potential, their wider use as anticancer immunostimulating drugs is hindered by their high systemic toxicity, caused primarily by low selectivity of their cytolytic action.^22–24^ A certain level of selectivity towards cancer cells was demonstrated and attributed to their cationic nature, as they interact preferably with the cancer cell membranes enriched by anionic lipids.^25,26^ However, the level of this selectivity may be insufficient *in vivo* to prevent lysis of healthy cells that results in adverse effects.

We have recently reported that the problems caused by high systemic toxicity and poor selectivity of the cytolytic peptides can be alleviated by converting them into photocontrolled analogues.^27^ These analogues can be designed to exist in two photoisomeric forms (photostates), one of which is less toxic and can be systemically administered, and the other, the active form, can be generated by irradiation with visible light (*e.g.* with red light) directly in tumors.

**Fig. 1:**
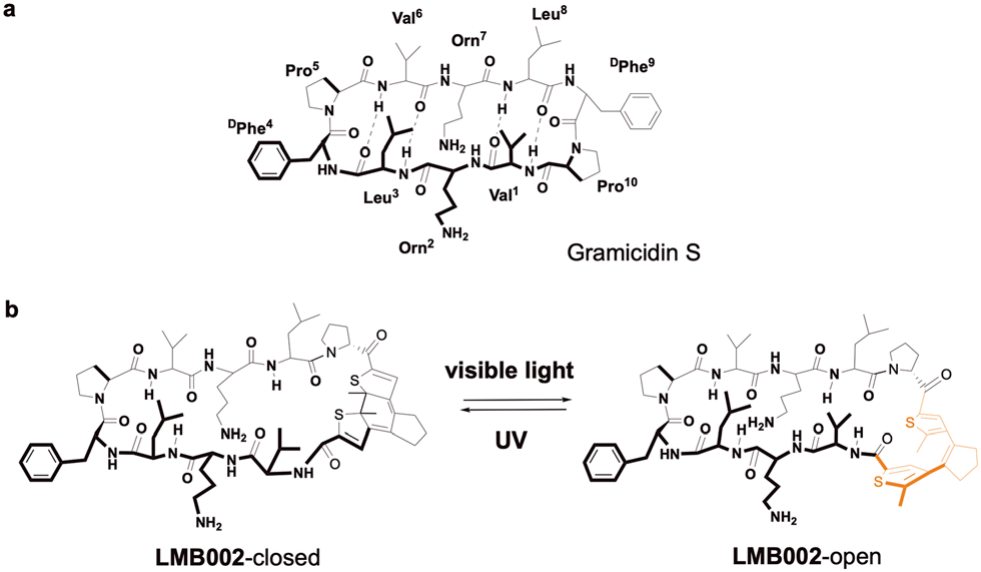
Prototype of peptide **GS** (a) and its photocontrollable analogue **LMB002** in two photostates (b). Photoisomerizable diarylethene fragment is highlighted in peach (“open” photoisomer) or grey (“closed” photoisomer) colours.

We documented indirect evidence that the anticancer activity of photocontrolled analogues of a cationic cytolytic peptide gramicidin S (**GS**, Fig. 1a) may have significant immunological components.^28^ Herein, we elaborate these observations and report the results of cell culture experiments that two such **GS**-derivatives, DAE-modified **LMB002** (Fig. 1b) and similar peptide **LMB033**,^29^ may indeed induce ICD and thus have immunogenic potential, which differs in the magnitude for the corresponding photoisomers.

## Results and discussion

We evaluated *in vitro* the ICD induction by **GS** and its photocontrolled analogues (in both photostates each) upon their exposure to selected cancer cell lines using three ICD hallmarks – ATP release, calreticulin exposure on the cell surface, and HMGB1 release. Prior to the investigation of the immunogenic potential of the peptides, their cytotoxic activity was evaluated. The membranolytic effect of **GS** itself that results in proliferation inhibition and eventual necrosis has been extensively investigated on prokaryotic^30^ and eukaryotic^31^ cells over the years. Guided by these data, we carried out a cytotoxic screening of our compounds in two cell culture formats – two-dimensional (monolayer) and three-dimensional (spheroid).

Five cancer cell lines of human (HeLa, cervical cancer; HepG2, hepatocellular carcinoma; MDA-MB-231, triple-negative breast cancer) and murine origin (LLC, Lewis lung carcinoma; 4T1, breast cancer) along with a cell line of non-cancerous origin, immortalized human embryonic kidney HEK-293, were chosen for the studies.

### Cytotoxicity of GS and its photoswitchable analogues in the adherent monolayer cell cultures

**Fig. 2:**
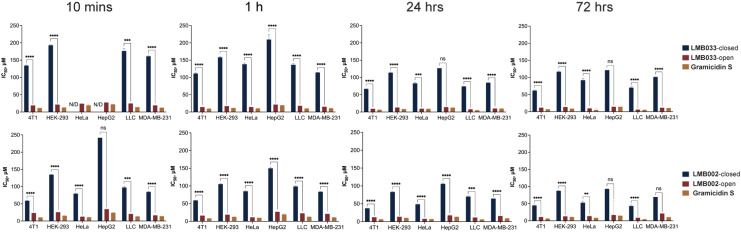
IC_50_ values deues determined for **LMB033**-closed, **LMB033**-open and gs (left panels) and **LMB002**-cl-closed, **LMB002**-open and **GS** (bottom panel) for 10 min, 1 h, 24 h and 72 h of incubation periods with six different cell lines grown as adherent monolayers (mean ± SEM (*n* = 4)). *p < 0.05, **p < 0.01, ***p < 0.001, ****p < 0.0001, ns: *p* > 0.05.

Screening of the cytotoxic activity of compounds in a two-dimensional (2D) format was designed in accordance with the previously obtained data^32^ for the HeLa cell line. The cytotoxic action of peptides was quantified in the 96-well plate format at four time points after adding compounds – at 10 min, 1 h, 24 h and 72 h. Staining with membrane-impermeable dye propidium iodide (PI) together with the DNA-intercalator Hoechst 33342 and subsequent automated fluorescence confocal imaging resulted in robust segregation of the fraction of non-viable necrotic cells (cells co-stained with both dyes) and determination of the concentration of half-maximum action, IC_50_ (ESI† Tables S1 and S2). The selected IC_50_ values for different incubation times are exemplified in Fig. 2.

Notably, the cell lines of murine origin (4T1 and LLC) appeared to be slightly more susceptible to the action of our compounds, whereas HEK-293 and HepG2 cells demonstrated resistivity. When analyzing the incubation time-dependent change of the closed/open isomer IC_50_ ratios for the monolayer-grown cell lines (Table 1), we noticed minor changes (**LMB002**: LLC; **LMB033**: HEK-293, HeLa, and MDA-MB-231), a decrease (**LMB002**: HeLa, HepG2, and MDA-MB-231; **LMB033**: 4T1 and HepG2) and an increase (**LMB002**: 4T1 and HEK-293; **LMB033**: LLC) in the cytotoxicity. This variation may stem from different membrane compositions and available cell surface, which are essential factors defining the sensitivity to cytolytic membrane-active peptides.^33,34^ The cytotoxicity of compounds develops over time of incubation with cells. Both **LMB002**-open and **LMB033**-open photoforms closely resemble parent **GS**, whereas both closed photoisomers demonstrated a markedly weaker activity. To assess the efficiency of photoswitching from the closed to open isomers, the ratios of IC_50_ values for the peptide photoforms were calculated (Table 1). These data suggest that **LMB033** has a better photoswitching efficiency (*i.e.* a larger difference in the cytotoxic activities of the photoisomers) than that of **LMB002**.

**Table 1:** IC_50_ ratios (IC_50_ of the closed photoisomer divided by IC_50_ of the open photoisomer) of photoswitchable peptides for different incubation times in cell monolayers

Cell line	10 min	1 h	24 h	72 h
For **LMB002**				
4T1	2.5	3.7	3.0	4.1
HEK-293	5.3	5.7	6.1	6.8
HeLa	6.3	7.4	6.3	3.9
HepG2	7.1	5.6	6.0	5.5
LLC	4.8	4.4	6.1	4.9
MDA-MB-231	5.2	4.0	4.1	3.3
For **LMB033**				
4T1	7.1	7.8	7.2	5.3
HEK-293	9.0	9.4	8.8	8.7
HeLa	—	9.7	8.8	9.8
HepG2	—	9.8	9.2	8.4
LLC	7.2	7.7	9.7	11.8
MDA-MB-231	8.4	7.6	8.5	8.8

### Cytotoxicity of GS and its photoswitchable analogues in spheroids

**Fig. 3:**
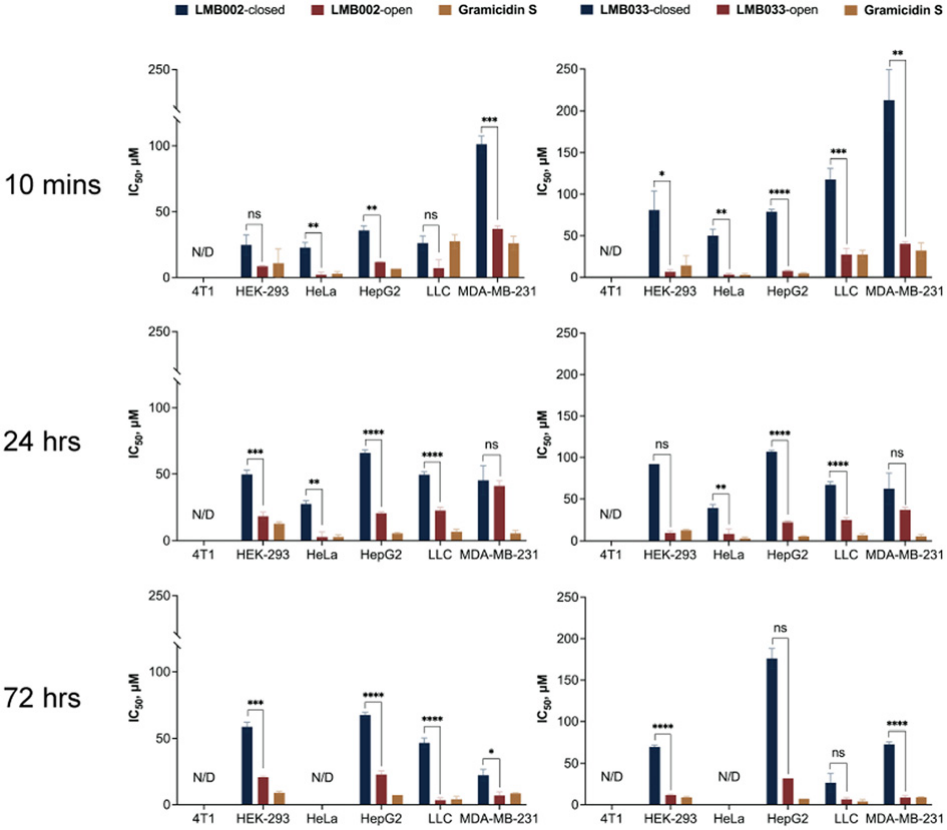
IC_50_ values determined for **LMB033**-closed, **LMB033**-open and **GS** (left panels) and **LMB002**-closed, **LMB002**-open and **GS** (right panels) for 10 min, 1 h and 72 h of incubation periods with six different cell lines grown as 3D cultures (mean ± SEM (*n* = 5)). *p < 0.05, **p < 0.01, ***p < 0.001, ****p < 0.0001, ns: *p* > 0.05.

For the experiments with the three-dimensional (3D) cultures, we used a 384-well plate format and applied the protocol introduced by Sirenko *et al.*,^35^ with a few modifications. First, we used the ultra-low adhesion plates to ensure reproducible spheroid formation. Second, we decided to co-stain the cells with calcein-AM to obtain more information on the processes occurring in spheroids upon the addition of cytolytic peptides. Calcein-AM stains metabolically active cells and helps to identify the fraction of necrotic cells that is essential for the accurate determination of the IC_50_ values (Fig. 3). Three time points of incubation of spheroids with the peptides (10 min, 24 h and 72 h) were chosen for the experiments. The IC_50_ values and the closed/open isomer activity ratios (Table 2) were calculated, as in the monolayer cell culture experiments.

**Table 2:** IC_50_ ratios (IC_50_ of the closed photoisomer divided by IC_50_ of the open photoisomer) of photoswitchable peptides for different incubation times in spheroids

Cell line	10 min	24 h	72 h
For **LMB002**			
4T1	N/D	N/D	N/D
HEK-293	2.9	2.7	2.8
HeLa	10.4	9.8	N/D
HepG2	3.0	3.2	3.0
LLC	3.7	2.2	13.4
MDA-MB-231	2.7	1.1	3.2
For **LMB033**			
4T1	N/D	N/D	N/D
HEK-293	12.3	9.6	5.9
HeLa	15.2	4.6	N/D
HepG2	10.4	4.7	5.5
LLC	4.3	2.7	4.0
MDA-MB-231	5.3	1.7	8.3

In line with the data obtained for monolayers, the closed forms of both **LMB002** and **LMB033** were less active than the corresponding open forms when added to spheroids. The open forms of **LMB002** and **LMB033** acted similarly to the parent compound **GS**. Half-activity values turned out to be smaller than those obtained for monolayer culture probably due to the presence of necrotic core in the spheroids that adds up to the number of necrotic cells.

**Fig. 4:**
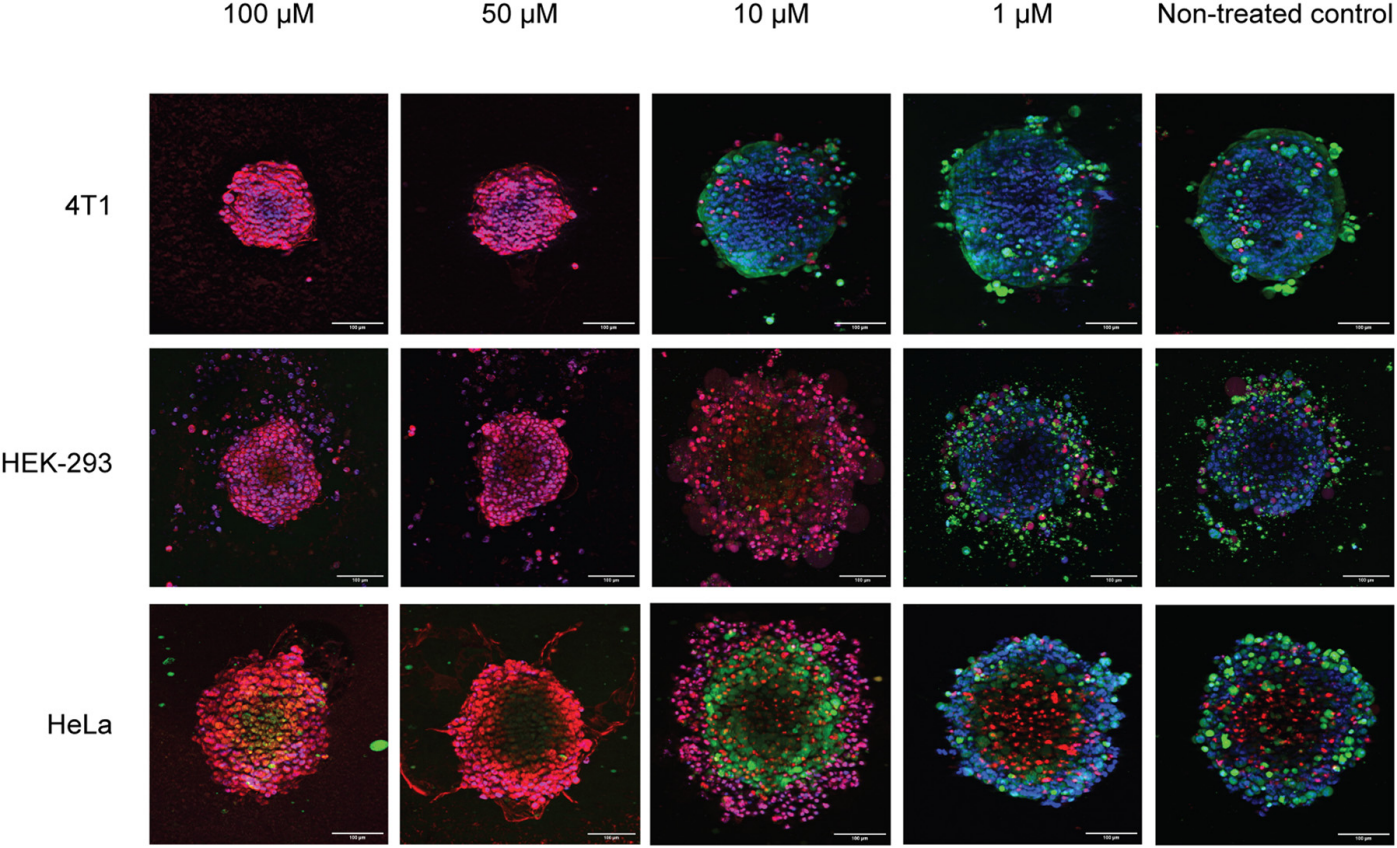
Maximum intensity projections of spheroids treated with different concentrations of **GS** for 24 h. Spheroids were stained with calcein-AM (green), PI (red) and Hoechst 33342 (blue). Scale bars are 100 μm.

**Fig. 5:**
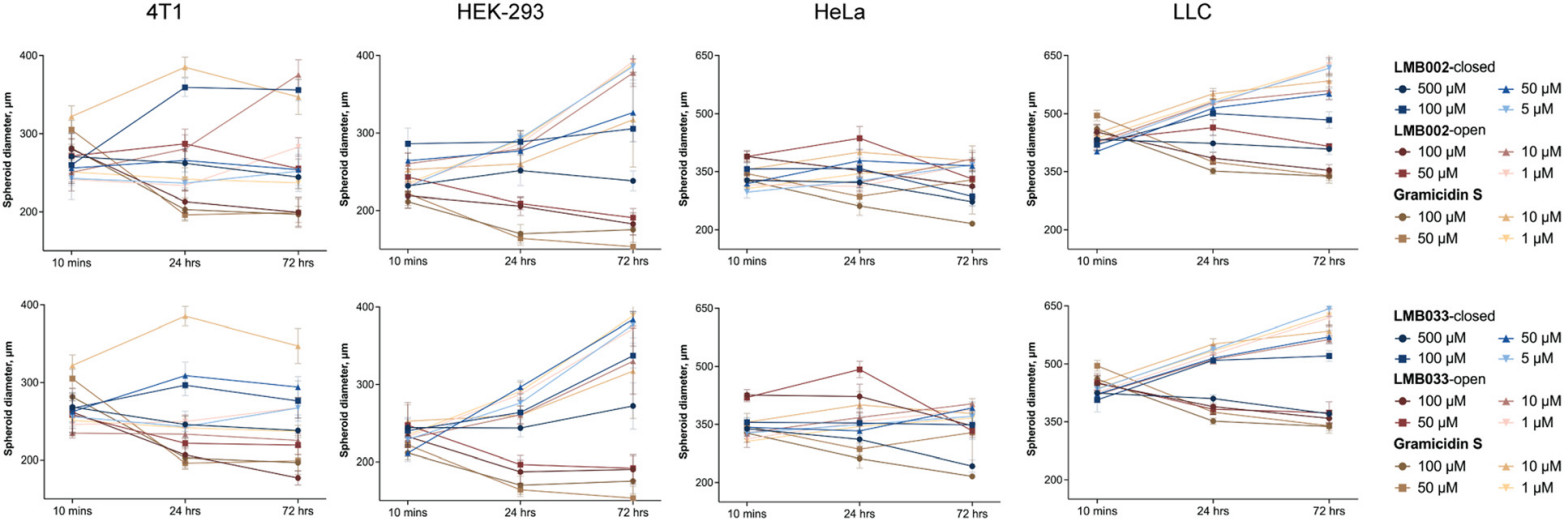
Change in the size of spheroids upon treatment with different concentrations of **LMB002**-closed, **LMB002**-open, **LMB033**-closed, **LMB033**-open and **GS** for 10 min, 24 h and 72 h. The data are presented as mean ± SEM (*n* = 5).

Additionally, a three-dimensional assay format provided insights into the ability of peptides to diffuse into spheroids' body and perturb its outer quiescent and proliferative zones (Fig. 4). Interestingly, in some cases, the compounds caused blebbing of cells that was described in the literature under similar conditions.^36^ Shrinkage or expansion of spheroids was evaluated by measuring the diameters of spheroids (Fig. 5).

We noted that the size of the 48 h spheroids was cell line-dependent (see Fig. 5, 10 min timepoint). The size ranged from 200–300 μm for HEK-293 to 400–500 μm for LLC cultures and were independent of the seeding density (see Experimental section) or species of cell origin, reflecting rather individual cell morphologies and growth kinetics. Upon exposure to the peptides for up to 72 h, we observed three types of spheroid size dynamics – continuous reduction of spheroid size (above IC_50_), further growth (HEK-293 and LLC), or preservation of the averaged spheroid diameters (4T1 and HeLa, below IC_50_). The size reduction (mostly pronounced when comparing 10 min *vs.* 24 h exposure to peptides) is related to the mechanism of cell killing,^37^ which is necrotic in essence and is accompanied by the outer layer cell detachment and, possibly, destruction of the cell–cell and cell-extracellular matrix contacts. In line with this, we also observed cell line-independent expansion of the spheroid diameters due to swelling, mostly pronounced at the peptide concentrations close to IC_50_.

### ATP release by eukaryotic cells in the presence of GS, LMB002 and LMB033

Adenosine triphosphate (ATP) is not only the critical energy carrier. It can also act as a neurotransmitter, mediating neuron–neuron and neuron-neuroglia communication in the brain. Leakage or regulated ATP release into extracellular space occurs in other tissues due to stress, hypoxia, ischemia, inflammation or cell injury. ATP is also involved in the complex interplay between inflammation, development of antitumor immunity and cancer immunotolerance.^38–40^ ATP signalling relies on the activation of purinergic receptors (P2Rs/P1Rs)^41^ that are broadly expressed on the surface of dendritic cells (DCs), macrophages, neutrophils and regulatory T cells. Glial and other immune cells possess ectonucleosidases (CD39+/CD73+), the enzymes that catalyse the formation of adenosine from ATP.^42,43^ ATP mainly promotes and adenosine inhibits the recruitment, maturation and differentiation of immune cells.^44^

According to the “Guidelines on Definition of ICD”,^13^ extracellular ATP is one of the main markers of ICD. As it has been recently discovered, established chemotherapeutics such as oxaliplatin, mitoxantrone^45^ and anthracyclines,^46^ as well as hypericin-based photodynamic therapy^47^ promote ATP release in the tumor microenvironment.

Treatment of cancer cells with lytic peptides was also reported to result in the ATP release,^20,21^ which is a good reason to believe that **GS** and our photoswitchable peptides may also demonstrate the same effect. To evaluate the ATP release, a luciferase-based luminescence assay was employed. The luminescence signal was acquired with an interval of 1 min between cycles for 1000 cycles after the addition of the compounds to the monolayer-grown cell cultures that allowed obtaining kinetic curves (see ESI† S16 and S18).

**Fig. 6:**
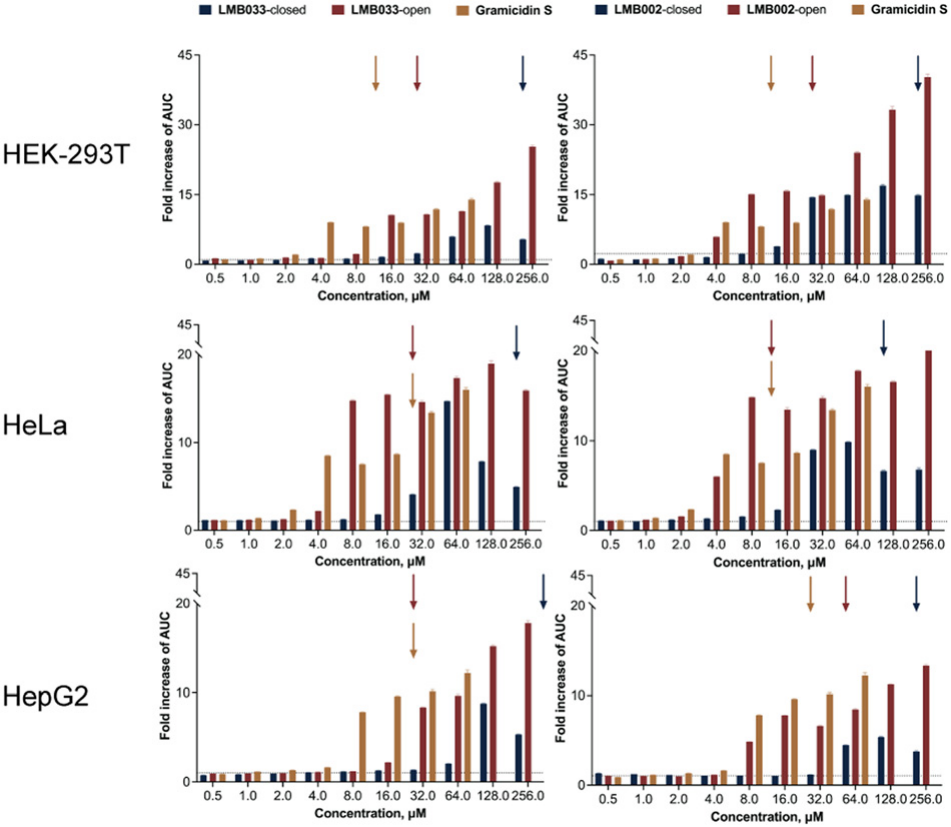
Area-under-the-curve (AUC) histograms for extracellular ATP released by cells upon treatment with **GS** and photoswitchable analogs (**LMB033**, left panels; **LMB002**, right panels) for 1000 min. Arrows signify the IC_50_ values for 10 min incubation measured earlier. The data are normalized to negative control (non-treated cells) as mean ± SEM (*n* = 3).

The calculation of the area under the curve (AUC) and normalization to non-treated control enabled the assessment of overall extracellular ATP in a dose-dependent manner for each tested compound and their comparison (Fig. 6). As expected from the cytotoxicity data, open photoisomers of the **GS** analogues and **GS** itself demonstrated greater ATP leakage into an extracellular space than the closed forms, with up to 20–40-fold increase in comparison to non-treated controls.

Another interesting detail is the dose-dependent differences between the compounds: for both open photoisomers and **GS**, the increase is rather dramatic with minor fluctuations at higher concentrations for the studied cell lines (HEK-293 making an exception), whereas for both closed photoisomers, the maximum is observed at 64 or at 128 μM with a detectable drop of the signal intensity at the following top concentration point. It may indicate the interaction of the closed isomers of peptides with luciferase/luciferin assay components, as is known for certain salts, detergents and peptides.^48,49^ We also analyzed ATP release taking the first derivatives of obtained curves (see ESI† S18 and S21): few extremes are usually observed that decay to zero within the first 30 min of monitoring. The most interesting finding is the fact that significant ATP release occurs at concentrations one or even two titration points lower than the corresponding IC_50_ values (denoted as arrows in Fig. 6). This observation suggests that ATP release at low concentrations is a regulated event, not a mere leakage that should occur at and above IC_50_ due to the membranolytic action of the peptides. This also demonstrates that a fraction of dead cells that constitutes less than 50% of the total cell population is sufficient to generate an immune system-activating ATP pool in the tumor microenvironment.

### CALR exposure on the surface of eukaryotic cells induced by GS and its photoswitchable analogues

Calreticulin (CALR) is an endoplasmic reticulum (ER)-residing protein that plays multiple important roles in all cell types.^50^ Inside the ER lumen, it acts as a lectin-like chaperone for glycoprotein post-translational modifications, owing to its high-capacity Ca^2+^-binding domain. CALR is the key ER regulator of Ca^2+^ homeostasis; in antigen-presenting cells, CALR takes part in the functioning of peptide-loading complex that ensures loading of antigens on MHC Class I molecules. When cells undergo ER stress, CALR is released from the ER lumen and translocated to the outer leaflet of the plasma membrane in a SNARE-dependent fashion (through exocytosis), where it starts functioning as an “eat-me” signal in complex with the oxidoreductase ERp57, which is also a part of the peptide-loading complex.^51^ It is noteworthy that ecto-CALR/ERp57 is a main phagocytic signal basally present on the surface of most human cancers and is exposed much less on the surface of normal, progenitor or stem cells.^52^ The immunogenicity of ecto-CALR is provided through its interaction with LRP1 or CD91 expressed on the surface of DCs. Once bound with LRP1, CALR stimulates phagocytosis and consequent maturation and activation of DCs, which further activate cytotoxic T lymphocytes, T helper cells or natural killer cells^53^ through IL- or TNF-mediated priming.^54^

As for many DAMPs, ecto-CALR-mediated signaling^21,55^ is a complex and tightly regulated process. First, ecto-CALR assists in cell migration and adhesion as well as angiogenesis, which are beneficial for tumor progression. Second, CALR translocation may differently shape the immunological outcome. For example, complete apoptosis inhibits immunogenic recognition of a dying cell,^56^ whereas post-apoptotic necrosis favors it.^57^ Third, CD47 counterbalances CALR activity; co-translocation of ERp57 and possibly other membrane-integrated proteins is required for the CALR exposure.^52,58^ In general, elevated levels of CALR expression are associated with a poor prognosis of cancer.^59^

**Fig. 7:**
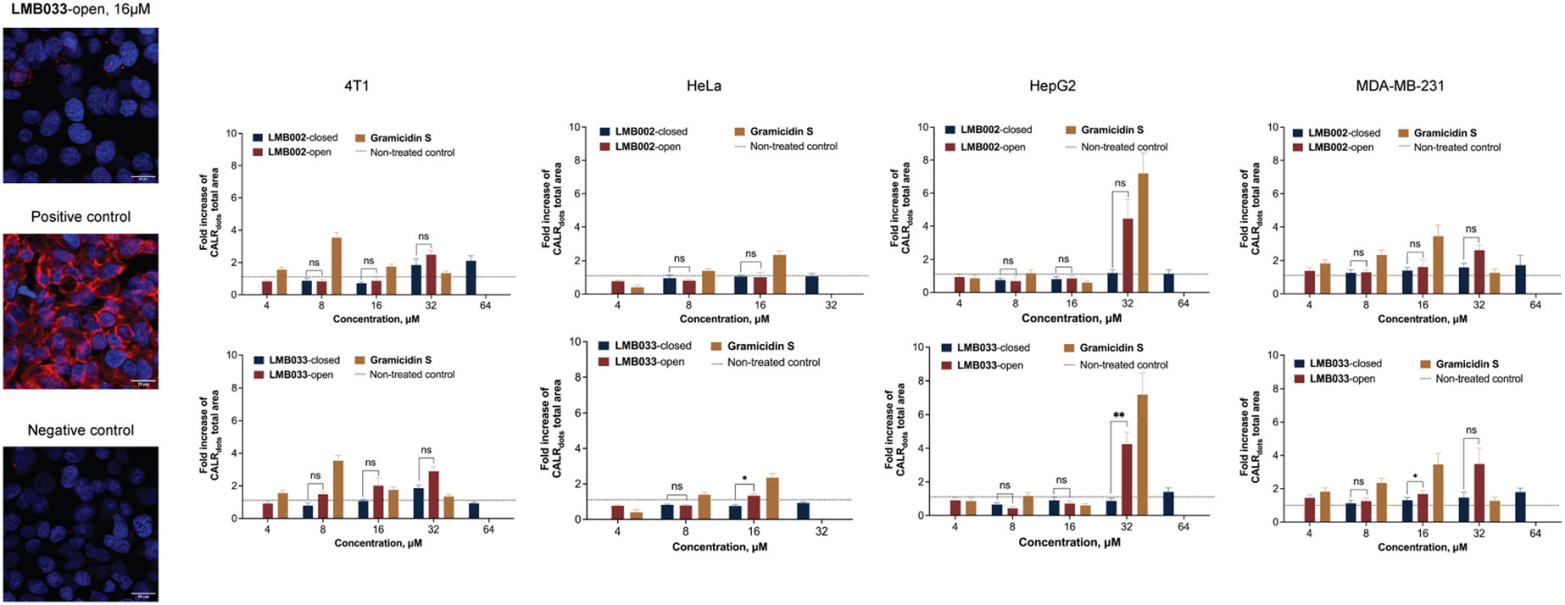
CALR exposure on the surface of cells upon treatment with compounds for 24 h. The data are normalized to negative controls (non-treated cells) as mean ± SEM (*n* = 2).

We performed fluorescence microscopy using an Alexa Fluor 647 anti-CALR antibody for quantifying the CALR exposure on the surface of cells. Confluent monolayer 4T1, HeLa, HepG2 and MDA-MB-231 cell cultures were incubated with GS and GS-derived peptides for 24 h followed by fixation, staining with the antibody and microscopic observation. The representative images obtained in our experiments are given in Fig. 7. Using the images, we calculated the total levels of the fluorescence signal per cell in each sample. The obtained data demonstrated the difference between the tested cell lines in CALR exposure in the chosen concentration range. While practically no enhancement in CALR exposure could be detected in the HeLa cell culture, a 4–7-fold enhancement is observed for HepG2 at 32 μM of **GS**, **LMB002**-open and **LMB033**-open. Then 8 and 16 μM of **GS** caused a 3-fold increase of CALR exposure in breast cancer cell lines 4T1 and MDA-MB-231. We noted no direct dose dependence of CALR release in our experiments. It may stem from the difference in the kinetics of CALR exposure. To examine the time dependence of the CALR exposure, cytofluorimetric experiments were conducted on the MDA-MB-231 cell line (Fig. 9) using an Alexa Fluor 647 anti-CALR antibody. In this experiment, 30 min, 3 h, 6 h and 24 h were the time points for monitoring the fraction of cells expressing CALR on their surface.

**Fig. 8:**
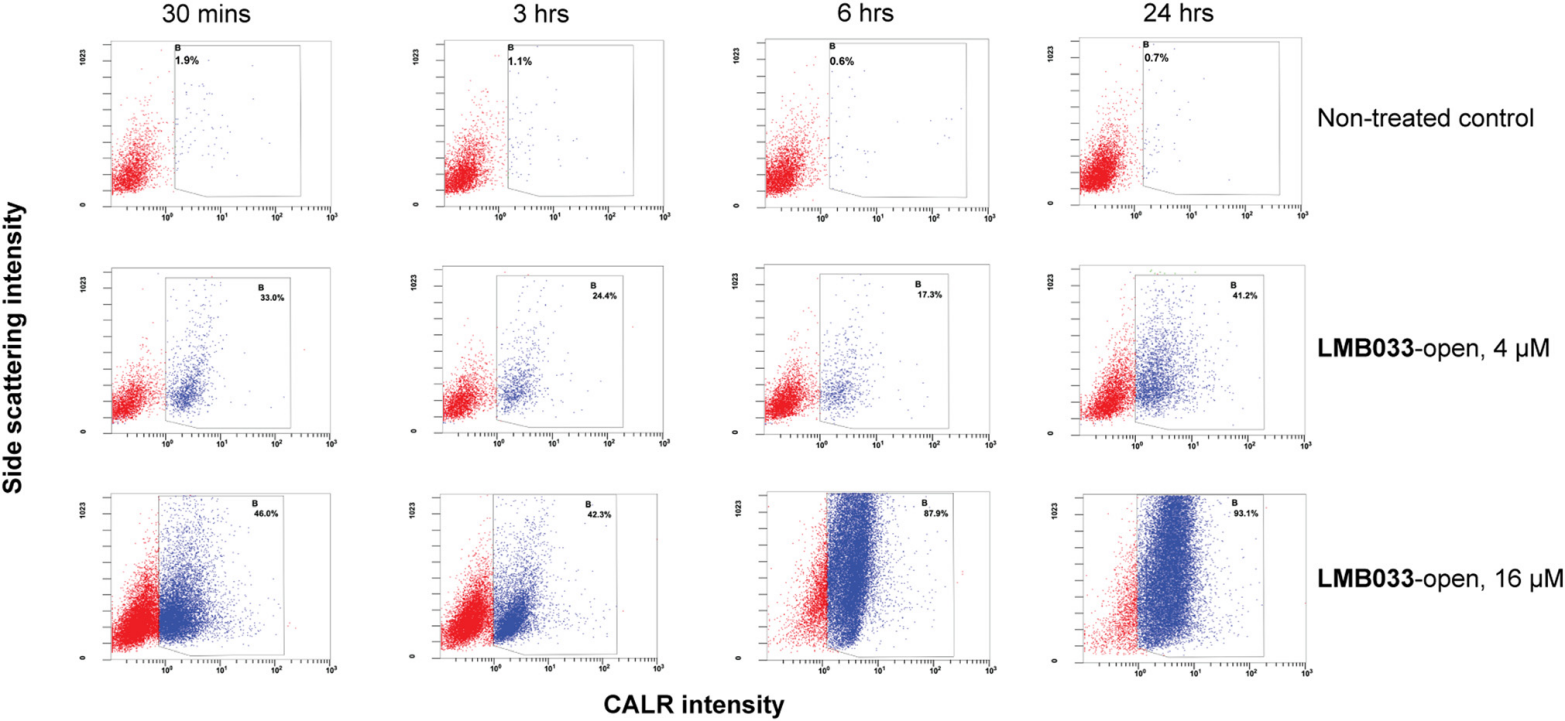
Representative scatter plots of MDA-MB-231 cells treated with **LMB033**-open at concentrations of 4 and 8 μM with antibody staining for membrane-bound CALR. The percentages on each graph illustrate the fraction of cells gated as CALR-positive.

In short incubation times, two sub-populations were observed in the samples (Fig. 8), with and without cellular membrane-bound CALR. However, at concentrations higher than IC_50_ (16 and 32 μM) after 6 h of incubation with **LMB033**-open (Fig. 8) and 3 h for **LMB002**-open or **GS** (see ESI† S22 and S23), all cells exposed CALR on their surface. These data indicated that it should take at least several hours for CALR to be fully exposed on the cell surface. This is different from the release kinetics of ATP, which was released from the cytosol of the cells with compromised plasma membranes almost immediately after the treatment with our peptides. Another interesting observation is the increase in side-scattering intensity with the concentration and time of incubation, which indicated an increase in cell granulation.

### HMGB1 release under treatment of eukaryotic cells with GS and its photoswitchable analogues

High mobility group box 1 (HMGB1) is a non-histone nuclear factor that participates in DNA bending over histone in nucleosomes and regulation of expression of several genes.^60^ Another important implication of HMGB1 is its action as an alarmin, an endogenous protein molecule involved in intercellular signalling,^61^ in particular, during necrosis. Though HMGB1 is passively released from the nuclei of necrotic cells,^62^ its consecutive mode of action relies on post-translational modifications, oxidation level, binding state and receptor type that it signals through.^63–65^

HMGB1 plays an exceptional role in cancer, impacting its progression and outcome. In general, cancer cells demonstrate elevated levels of HMGB1 expression that is associated with poor prognosis.^66–68^ HMGB1 sustains tumour growth and metastasis, favouring autophagy that prevents harbouring of tumour antigens by antigen-presenting cells.^69^ However, treatment with anthracyclines,^70,71^ oncolytic peptides,^21,55^ and vaccines,^72^ that elicits an immune response, is unambiguously proven to be accompanied by HMGB1 release. Such diverse effects of HMGB1 may be explained by the polycationic nature of this protein and its unique ability to interact with different biopolymers (cytokines, nucleic acids and lipopolysaccharides) and different receptors.^73^ Two major receptor types for HMGB1 are the receptor for advanced glycation end products (RAGE) and Toll-like receptors (TLRs) – both expressed on the surface of DCs, monocytes, macrophages, neutrophils and other immune cells. Interestingly, signalling *via* both RAGE and TLRs may either promote angiogenesis and metastasis or facilitate maturation, activation and recruitment of different types of immune cells that initiate downstream cascades of cross-activation.^74^ Despite its opposite roles in cancer progression, the study of HMGB1 release upon treatment with a given compound is helpful in understanding its immunogenic potential.

**Fig. 9:**
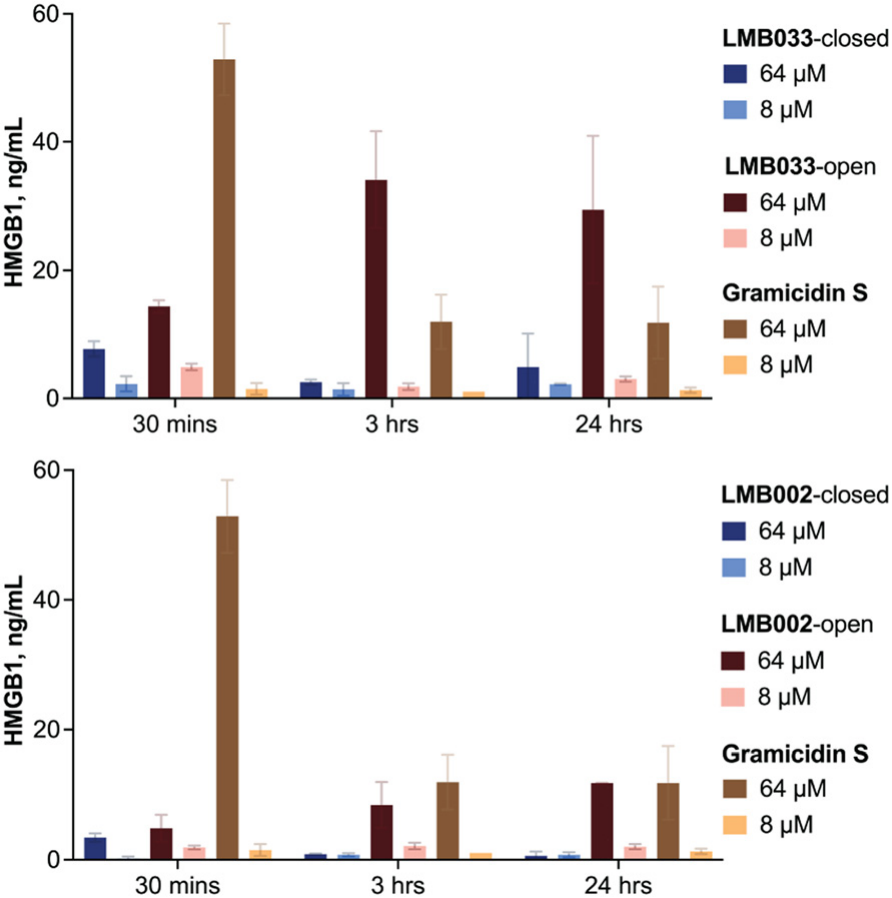
HMGB1 detected in the supernatant after incubation of HepG2 cells with the peptides for 30 min, 3 h and 24 h. The data are presented after interpolation as mean ± SEM (*n* = 3).

HepG2 cells were treated with our peptides at concentrations of 8 and 64 μM for 30 min, 3 h and 24 h. HMGB1 was quantified (using a calibration curve) by a bioluminescence assay in the supernatant. The results are shown in Fig. 9.

As can be seen in Fig. 9, the fastest release of HMGB1 was induced by **GS** at a concentration of 64 μM. In this experiment, 50 ng mL^−1^ of HMGB1 was detected in the supernatant after 30 min post-treatment. However, the concentration of the protein in the supernatant decreased at 3 h and remained constant up to 24 h (10 ng mL^−1^), suggesting that after the initial spike, continuous release of HMGB1 and its degradation occur over time (half-time of degradation of HMGB1 in a 10% FBS-supplemented medium is around 7 h).^75^ A slightly different picture is observed for open photoforms of both the photocontrolled peptides: the maximum release is reached at a 3 h time point and retained at 24 h. However, the most striking difference between **LMB002**-open and **LMB033**-open was observed upon treatment at 64 μM concentrations: **LMB033**-open-treated cells released 15–35 ng mL^−1^ HMGB1 in the medium, while with **LMB002**-open, the maximum concentration was lower, 10 ng mL^−1^. The closed photoforms also demonstrated quite a noticeable difference: **LMB033** (64 μM) gave a spike at 30 min with 10 ng mL^−1^ of protein released, whereas **LMB002**-closed did not trigger the HMGB1 release. No significant induction of HMGB1 release was observed after treatment with 8 μM (sub-IC_50_) of any of the tested compounds.

## Conclusions

Systematic cytotoxicity assessments, carried out in this study on 2D (adherent monolayer) and 3D (spheroid) cultures of cells of human and murine origin, demonstrated fast-onset low micromolar activity for **gramicidin S** and its diarylethene-derived photocontrolled analogues **LMB002** and **LMB033** in the open photoforms. In line with our previous results, activity of the closed forms of the **GS** analogues was 2–12 times lower than that of the open forms. Moderate cell-specificity was observed for the studied compounds, especially pronounced on spheroids. As a general trend, lower activity was observed for the cells of non-cancerous origin; this can be explained by the different membrane composition of the cells, assuming that the primary mechanism of action of the compounds is the cellular membrane destabilisation.

All three markers assayed in this work (ATP, calreticulin, and HMGB1) in cell cultures after treatment with the studied compounds demonstrated the level and kinetics typical for ICD. A clear indication of ICD was observed even at sub-IC_50_ – an important observation for planning future *in vivo* research. As might be expected from the cytotoxicity assay results, the first signs of ICD were observed at lower concentrations of the open photoisomers of **LMB002** and **LMB033** than those of the corresponding closed photoisomers. Comparing the photocontrolled analogues, it appears that **LMB033** has superior characteristics of the cytotoxicity level in the open-form, higher open-form/closed-form cytotoxicity ratio, as well as the more pronounced potential to induce ICD, compared to **LMB002**.

## Experimental section

### Peptides

The peptides **GS**, **LMB002**, and **LMB033** were the products of solid-phase peptide synthesis obtained at Lumobiotics. Photoswitchable peptides were prepared as pure (>95%, reverse-phase high-performance liquid chromatography) open and closed photoisomers and were kept in the darkness as frozen stock solutions in dimethyl sulphoxide. The details of peptide synthesis, procedures for preparing pure photoisomers, structures^76,77^ and photophysical characterisation^78^ have been previously published.

### Cell lines

A 4T1 cell line (CRL-2539, ATCC) was cultivated in an RPMI-1640 medium (RPMI-A-P10, Capricorn Scientific) supplemented with 1% penicillin–streptomycin (P0781, Sigma) and 10% heat-inactivated fetal bovine serum (FBS, F7524, Merck) at 37 °C, 5% CO_2_, 95% RH. HeLa (CCL-2, ATCC), HEK-293 (CRL-1573, ATCC), HepG2 (HB-8065, ATCC), LLC (CRL-1642, ATCC) and MDA-MB-231 (ACC 732, DSMZ) cell lines were cultivated in Dulbecco's modified Eagle's medium (DMEM) (SH30003.04, Cytiva) supplemented with 1% penicillin–streptomycin, 1% l-glutamine (G7513, Merck) and 10% FBS at 37 °C, 5% CO_2_, 95% RH. For ATP detection and HMGB1 release experiments, HeLa (CCL-2, ATCC), HepG2 (HB-8065, ATCC), and HEK-293 T (CRL-3216, ATCC) were maintained in DMEM (41966-029, Thermo Fisher) supplemented with 1% penicillin–streptomycin (CP23-6514, Capricorn Scientific), 1% l-glutamine (25030081, Thermo Fisher) and 10% FBS (10270106, Thermo Fisher) at 37 °C, 5% CO_2_, 95% RH.

### Cytotoxicity

Essential experimental details have been previously published by us.^79^

### Two-dimensional cultures (monolayer)

The cells were maintained in a culture till 70–80% confluence was reached and then seeded in a 96-well black-wall transparent-bottom plate (6055302, Revvity) with 200 μL of appropriate medium per well. The seeding density has been previously determined in the optimization experiments for each cell line: 3500 cells per well for 4T1; 20 000 cells per well for HEK-293; 20 000 cells per well for HeLa; 25 000 cells per well for HepG2; 8000 cells per well for LLC; 15 000 cells per well for MDA-MB-231. After 24 h incubation at 37 °C, the medium was removed and 100 μL of fresh medium was added. Two-fold serial dilution of compounds (256–0.5 μM, 11 points for closed forms of photoswitchable peptides; 128–0.25 μM, 11 points for open forms; 64–0.5, 8 points for GS) in the medium was prepared and dispensed in 100 μL per well. The plates were incubated with compounds for 10 min, 1 h, 24 h and 72 h. The staining solution consisting of 25 μM of Hoechst 33342 (62249, Thermo Fisher), 5 μM of propidium iodide (J66764-MC, Thermo Fisher) or ethidium homodimer-1 (E1169, Thermo Fisher), with 10% FBS in DPBS (21600044, Thermo Fisher) was prepared and added to wells in a volume of 50 μL for 20 min. Fluorescence images were acquired using an INCell Analyzer 6500 HS automatic imaging system with 20× objective lens (NA = 0.45) and 16 fields of view (FOV) per well. The numerical data were obtained using INCarta 1.13 and then analysed using GraphPad Prism 10.

### Three-dimensional cultures (spheroids)

The cells were maintained identically to a 2D cell culture protocol. After counting, the cells were seeded in 50 μL of medium in an ultralow attachment U-bottom black-wall 384-well plate (3830, Corning). The seeding density was chosen to obtain spheroids after 48 h of incubation that are compact enough to fit into 20× objective field of view (<800–1000 μm in diameter) after incubation with compounds. We seeded 2000 cells per well for 4T1 and 1000 cells per well for HEK-293, HeLa, HepG2, LLC and MDA-MB-231. After seeding, the plate was centrifuged at 40 g for 30 s and additionally shaken at 250 rpm for 1 min to shake all the cells off the walls of a well. After 48 h, the compounds were dispensed in 20 μL of medium (500, 100, 50, 5 and 1 μM for closed peptide photoforms; 100, 50, 10, 1 and 0.5 μM for open photoforms and **GS**). At 10 min, 24 h and 72 h post addition of compounds, the staining solution consisting of 112.5 μM of Hoechst 33342, 4.5 μM of calcein-AM (C1430, Thermo Fisher), 13.5 μM of propidium iodide and 10% of FBS in DPBS was added in 20 μL for 2 h. The fluorescent imaging was conducted in the confocal mode with a 20× objective lens and z-stack of 12–15 images with a 10 μm step. The collected data were processed using INCarta 1.13 and analyzed using GraphPad Prism 10.

### ATP release

The cells were seeded (10 000 cells per well for HeLa; 25 000 cells per well for HepG2, 20 000 cells per well for HEK-293T) in 200 μL of DMEM complete in a 96-well black-wall transparent-bottom plate (655090, Greiner). Next day, the medium was aspired and 100 μL of DPBS (14190144, Thermo Fisher) was used for rinsing the cells. Then, 100 μL of Leibovitz L-15 medium (21083027, Thermo Fisher) supplemented with 10% FBS was added. Two-fold serial dilution (256–0.5 μM, 11 points for closed photoforms; 128–0.25 μM, 11 points for open photoforms; 64–0.5, 8 points for **GS**) was prepared in a Leibovitz L-15 medium in an intermediate plate. A RealTime-Glo extracellular ATP assay (GA5011, Promega) reagent was diluted with a Leibovitz L-15 medium to acquire a 4× solution. It was warmed up to room temperature and dispensed in 50 μL per well. After that the plate was filled with tested compound solutions from the intermediate plate (50 μL per well), sealed with a transparent film (GZ-13024-02, Excel Scientific) and put immediately into a spark multimode microplate reader (Tecan) preheated to 37 °C. Luminescence signal was collected over the visible light range (360–700 nm) for 1000 min (cycle interval: 1 min, integration time: 500 ms). The data were analyzed using GraphPad prism 10.

### CALR exposure

#### Fluorescence microscopy

The cells were seeded (10 000 cells per well for 4T1; 20 000 cells per well for HeLa and MDA-MB-231; 30 000 cells per well for HepG2) in 200 μL of appropriate medium in a 96-well black-wall transparent-bottom plate (6055302, Revvity). After 24 h incubation, the medium was aspired and 100 μL of fresh medium was added. The compounds (64, 32, 16 and 8 μM for closed photoforms; 32, 16, 8 and 4 μM for open photoforms and **GS**) along with the negative (1% DMSO) control were prepared in DMEM and dispensed in 100 μL per well for 24 h. Then 50 μL of 20% paraformaldehyde (28908, Thermo Fisher) was added for 15 min for fixation. The plate was washed 3 times with PBS using a Microplate Washer EL 450 (BioTek). For the positive control, wells with untreated cells were permeabilized by incubating with 0.1% Triton X-100 (93443, Merck) in PBS for 15 min. After washing, the fixed cells were incubated in 50 μL per well of solution containing 1% BSA, 0.3 M glycine, and 0.1% Tween 20 in PBS for 30 min at RT. The solution was aspired and the staining solution (1/1000 Recombinant Alexa Fluor-647 anti-calreticulin antibody (ab196159, Abcam), 1/4000 Hoechst 33342, 1% BSA, 0.1% Tween 20 in PBS) was dispensed in 100 μL per well for 30 min. The plate was washed again and sealed with an aluminum film. The imaging was performed using a 40× objective lens (NA = 0.95) with subsequent analyses using INCarta Software 1.13 and GraphPad Prism 10.

#### Cytofluorimetric measurements

MDA-MB-231 cells were seeded at a density of 100 000 cells per well of a 12-well cell culture plate (Z707775, Merck) in 1 mL of RPMI-1640 complete medium and incubated at 37 °C for 24 h. The medium was aspired and 900 μL of fresh medium was added. Serial dilution of compounds (40, 80, 160 and 320 μM) was performed in the same medium and dispensed to the assay plate (100 μL per well). The cells were incubated with compounds for 30 min, 3 h, 6 h and 24 h before cytofluorimetric analysis. For the analysis, the medium was removed, and the cells were washed with DPBS, harvested with Versene 1 : 5000 and then gently detached manually using a sterile cell scraper. Obtained cell suspensions were centrifuged for 10 min at 1500 rpm. Supernatants were removed and replaced with 100 μL DPBS. Then, 20 μL of Alexa Fluor-647 anti-calreticulin antibody (ab196159, Abcam) was diluted in PBS (1 : 100) with the addition of 1% sodium azide to each sample. The samples were mixed and incubated for 20 min in the darkness at RT. Then, 1 mL of PBS was added to each sample, and the samples were centrifuged for 7 min at 1500 rpm. The supernatant was removed and replaced with 500 μL of PBS with resuspending the pellet. Then, 5 min before the cytofluorimetric analysis, 5 μL of 40 μM 7-aminoactinomycin D (7-AAD) (00-6993, Thermo Fischer) was added to all samples to check the cell viability. The measurement was performed using a Navios EX flow cytometer (Beckman Coulter) with FL3 (ex. 488, em. 614/20) and FL6 (ex. 638, em. 660/20) for the detection of 7-AAD and AlexaFluor-647 fluorophores correspondingly.

#### HMGB1 release

For this, 25 000 cells per well of HepG2 were seeded in 200 μL of complete DMEM in a 96-well black-wall transparent-bottom plate (655090, Greiner). After 24 h, the medium was aspired, and 100 μL of DPBS was added for rinsing and then aspired. Then, 100 μL of Leibovitz L-15 medium that contained 10% FBS was added. After that, 2× concentrated solutions of tested compounds (64 and 8 μM, triplicates for each time point) were prepared in the same Leibovitz L-15 medium in an intermediate plate and dispensed in a volume of 100 μL to the cell-containing plate. The calibration curve was constructed using a HMGB1 stock solution (1000–0.24 ng mL^−1^, 4-fold dilution, 7 points, *n* = 2) from Lumit HMGB1 (Human/Mouse) immunoassay kit (W6110, Promega) and dispensed in a 96-well white-wall transparent-bottom working plate (353377, Falcon). After 30 min, 3 h and 24 h, aliquots from the intermediate plate (tested compounds) were transferred to the working plate. Antibody complexes (LgBiT : SmBiT : Leibovitz L-15 medium = 1 : 1 : 50) were prepared and dispensed to the working plate to result in a test well content-to-antibody complex ratio of 4/1. The plate was shaken for 1 min and incubated for 1 h at room temperature. Detection substrate B (W6110, Promega) was diluted in a detection buffer (W6110, Promega) in a ratio of 1/20 and dispensed to the working plate in 1 : 4 proportion to the present solution. The luminescence signal was collected throughout the visible range (360–700 nm). The data were analyzed using GraphPad Prism 10.

## Author contributions

KH, OH, NK, VK, ASU, SA, and IVK performed methodology and formal analysis; KH, IS, OH, SK, and SA performed investigation; KH and IVK prepared the original draft; all the authors reviewed and edited the manuscript; NK, PB, ASU, SA, and IVK performed supervision.

## Conflicts of interest

IVK, SA, and ASU are inventors on the issued patent family: “Peptidomimetics possessing photocontrolled biological activity” (WO2014127919 [A1], EP2958934 [B1], US9481712 [B2], UA113685 [C2]) licensed to Lumobiotics GmbH. IVK and SA are founders and shareholders of Lumobiotics GmbH. IVK is a scientific advisor, and KH, VK and PB are employees of Enamine LLC. The authors have no other relevant affiliations or financial involvement with any organization or entity with a financial interest in or financial conflict with the subject matter or materials discussed in the publication apart from those disclosed.

## Supplementary Material

MD-016-D5MD00075K-s001

## Data Availability

Data for this article, including the raw data for all the bioassays described in the manuscript in graphical and table forms, are available at the public repository Zenodo (https://doi.org/10.5281/zenodo.14717738) and are provided in the ESI.†
