# Supplementary material for: In vitro evaluation of the immunogenic potential of gramicidin S and its photocontrolled analogues
Source: RSC Med Chem. 2025 Apr 10;16(8):3456–68. doi: 10.1039/d5md00075k (PMC12013366; doi:10.1039/d5md00075k)

Supplementary Information (SI) for RSC Medicinal Chemistry.
This journal is © The Royal Society of Chemistry 2025

Supplementary information (SI)

In Vitro Evaluation of Immunogenic Potential of Gramicidin S and its Photocontrolled Analogues

Kateryna Horbatok, Iryna Semchuk, Oleksandr Horbach, Natalia Khranovska, Viktoriia Kosach, Petro Borysko, Serhii Koniev, Anne S. Ulrich, Sergii Afonin and Igor V. Komarov

Table of Contents

Table S1. Cytotoxic activity of LMB033-closed, LMB033-open and gramicidin S monitored in two-dimensional assay. 3

Table S2. Cytotoxic activity of LMB002-closed, LMB002-open and gramicidin S monitored in two-dimensional assay. 4

Table S3. Cytotoxic activity of LMB033-closed, LMB033-open and gramicidin S monitored in three-dimensional (spheroid) assay 5

Table S4. Cytotoxic activity of LMB002-closed, LMB002-open and gramicidin S monitored in three-dimensional (spheroid) assay. 6

Figure S1. Dose-dependent curves for gramicidin S and its photocontrolled analogues tested on 4T1 cell line in two-dimensional format. 7

Figure S2. Dose-dependent curves for gramicidin S and its photocontrolled analogues tested on HEK-293 cell line in two-dimensional format. 8

Figure S3. Dose-dependent curves for gramicidin S and its photocontrolled analogues tested on HeLa cell line in two-dimensional format. 9

Figure S4. Dose-dependent curves for gramicidin S and its photocontrolled analogues tested on HepG2 cell line in two-dimensional format. 10

Figure S5. Dose-dependent curves for gramicidin S and its photocontrolled analogues tested on LLC cell line in two-dimensional format. 11

Figure S6. Dose-dependent curves for gramicidin S and its photocontrolled analogues tested on MDA-MB-231 cell line in two-dimensional format. 12

Figure S7. Dose-dependent curves for gramicidin S and its photocontrolled analogues tested on HEK-293 cell line in three-dimensional format. 13

Figure S8. Dose-dependent curves for gramicidin S and its photocontrolled analogues tested on HeLa cell line in three-dimensional format. 14

Figure S9. Dose-dependent curves for gramicidin S and its photocontrolled analogues tested on HepG2 cell line in three-dimensional format. 15

Figure S10. Dose-dependent curves for gramicidin S and its photocontrolled analogues tested on LLC cell line in three-dimensional format. 16

Figure S11. Dose-dependent curves for gramicidin S and its photocontrolled analogues tested on MDA-MB-231 cell line in three-dimensional format. 17

Figure S12. Kinetic curves of ATP release monitored for 1000 minutes after addition of gramicidin S and its photocontrolled analogues (run 1). 18

Figure S13. First derivatives of kinetic curves of ATP release monitored for 1000 minutes after addition of gramicidin S and its photocontrolled analogues (run 1). 19

Figure S14. Kinetic curves of ATP release monitored for 1000 minutes after addition of gramicidin S and its photocontrolled analogues (run 2). 20

Figure S15. Area-under-curve (AUC) histrograms of ATP release monitored for 1000 minutes after addition of gramicidin S and its photocontrolled analogues (run 2). 21

Figure S16. First derivatives of kinetic curves of ATP release monitored for 1000 minutes after addition of gramicidin S and its photocontrolled analogues (run 2). 22

Figure S17. Scatterplots of MDA-MB-231 cells treated with LMB033-open 23

Figure S18. Scatterplots of MDA-MB-231 cells treated with LMB002-open 24

Figure S19. Scatterplots of MDA-MB-231 cells treated with gramicidin S 25

Table S1. Cytotoxic activity of LMB033-closed, LMB033-open and gramicidin S monitored in two-dimensional assay. IC_{50} values are presented as absolute value ± SEM in µM (n=4).

	**LMB033**-closed				**LMB033**-open			
Cell line	10 mins	1 h	24 hrs	72 hrs	10 mins	1 h	24 hrs	72 hrs
4T1	134.2 ± 1.5	111.5 ± 1.8	66.5 ± 0.8	61.6 ± 1.7	18.8 ± 0.3	14.3 ± 0.1	9.2 ± 0.2	11.6 ± 0.3
HEK-293	193.3 ± 2.2	158.3 ± 1.8	113.8 ± 1.8	116.8 ± 2.3	21.4 ± 0.3	16.9 ± 0.3	13.0 ± 0.1	13.5 ± 0.7
HeLa	N/D	138.3 ± 3.5	83.0 ± 3.0	92.0 ± 5.8	24.1 ± 0.5	14.3 ± 0.3	9.4 ± 0.2	9.4 ± 1.6
HepG2	N/D	209.7 ± 14.1	126.8*	121.4*	27.3 ± 0.4	21.4 ± 0.7	13.8 ± 0.5	14.5*
LLC	176.7 ± 6.8	136.5 ± 4.2	73.8 ± 1.2	70.5 ± 2.7	24.5 ± 1.1	17.8 ± 2.0	7.6 ± 0.2	6.0 ± 0.2
MDA-MB-231	161.3 ± 1.8	114.1 ± 1.0	84.6 ± 1.0	101.3 ± 0.7	19.2 ± 0.2	15.1 ± 0.2	9.9 ± 0.3	11.5 ± 0.1
	**Gramicidin S**							
Cell line	10 mins	1 h	24 hrs	72 hrs				
4T1	11.3 ± 0.2	9.7 ± 0.2	6.4 ± 0.4	6.9 ± 0.1				
HEK-293	12.9 ± 0.4	11.6 ± 0.1	8.3 ± 0.2	8.4 ± 0.7				
HeLa	19.3 ± 2.9	10.3 ± 0.6	8.9 ± 0.5	4.7 ± 3.5				
HepG2	22.0 ± 0.4	19.3 ± 0.5	12.6 ± 0.2	14.1*				
LLC	14.2*	9.9 ± 1.0	4.9 ± 0.3	5.2 ± 0.7				
MDA-MB-231	12.3 ± 0.3	10.8 ± 0.2	9.7 ± 0.4	10.4 ± 0.2				

N/D (non-defined) marks cases where IC_{50} activity falls out of tested concentration range.

* SEM was not defined due to specifics of data analysis methodology.

Table S2. Cytotoxic activity of LMB002-closed, LMB002-open and gramicidin S monitored in two-dimensional assay. IC_{50} values are presented as absolute value ± SEM in µM (n=4).

	**LMB002**-closed				**LMB002**-open			
Cell line	10 mins	1 h	24 hrs	72 hrs	10 mins	1 h	24 hrs	72 hrs
4T1	58.7 ± 0.5	59.1 ± 0.7	37.1 ± 0.2	44.6 ± 1.4	23.3 ± 0.3	16.1 ± 0.1	12.3 ± 0.2	10.8 ± 0.4
HEK-293	134.5 ± 1.3	105.1 ± 1.7	83.0 ± 1.5	87.3 ± 1.8	25.5 ± 0.8	18.6 ± 0.5	13.6 ± 0.3	12.9 ± 0.1
HeLa	79.3 ± 1.8	84.9 ± 1.5	48.4 ± 1.0	52.6 ± 4.6	12.6 ± 0.5	11.4 ± 0.3	7.7 ± 0.7	13.6 ± 1.1
HepG2	241.9*	150.0 ± 3.3	106.0 ± 2.0	92.6 ± 1.2	34.3 ± 0.8	26.7 ± 0.6	17.6 ± 2.3	16.9*
LLC	97.6 ± 3.5	98.5 ± 1.7	70.3 ± 1.9	43.3 ± 2.7	20.5 ± 0.4	22.6 ± 0.3	11.5 ± 0.2	8.8 ± 0.4
MDA-MB-231	84.6 ± 1.1	83.5 ± 1.3	64.3 ± 0.3	69.5*	16.4 ± 0.5	21.0 ± 0.3	15.7 ± 0.1	21.1 ± 0.2

	**Gramicidin S**			
Cell line	10 mins	1 h	24 hrs	72 hrs
4T1	10.3 ± 0.2	8.3 ± 0.1	6.4 ± 0.2	6.2 ± 0.5
HEK-293	15.4 ± 0.1	12.7 ± 0.2	10.4 ± 0.1	11.1 ± 0.1
HeLa	10.9 ± 0.3	9.8 ± 0.2	6.8 ± 0.7	8.3 ± 0.4
HepG2	24.0 ± 0.9	19.6 ± 1.5	13.4 ± 0.3	15.0*
LLC	13.1 ± 0.8	12.7 ± 0.7	6.0 ± 0.2	4.4 ± 0.2
MDA-MB-231	13.9 ± 0.2	11.0 ± 0.1	9.1 ± 0.3	10.4 ± 0.1

* SEM was not defined due to specifics of data analysis methodology.

Table S3. Cytotoxic activity of LMB033-closed, LMB033-open and gramicidin S monitored in three-dimensional (spheroid) assay. IC_{50} values are presented as absolute value ± SEM in µM (n=5).

	**LMB033**-closed			**LMB033**-open			**Gramicidin S**		
Cell line	10 mins	24 hrs	72 hrs	10 mins	24 hrs	72 hrs	10 mins	24 hrs	72 hrs
4T1	N/D	N/D	N/D	N/D	N/D	N/D	N/D	N/D	N/D
HEK-293	81.0 ± 22.9	92.1*	69.8 ± 2.2	6.6 ± 2.6	9.6 ± 2.3	11.9 ± 1.0	14.3 ± 11.6	12.9 ± 1.1	9.0 ± 1.1
HeLa	50.1 ± 8.0	39.0 ± 4.4	N/D	3.3 ± 1.7	8.5 ± 5.9	N/D	2.9 ± 1.5	2.9 ± 1.6	N/D
HepG2	79.1 ± 2.9	106.8 ± 1.6	176.2 ± 12.2	7.6 ± 0.9	22.5 ± 1.1	31.9*	4.8 ± 0.8	5.5 ± 0.8	7.2*
LLC	117.8 ± 13.4	67.0 ± 3.8	26.6 ± 11.3	27.4 ± 7.5	24.6 ± 2.9	6.7 ± 2.2	27.4 ± 5.2	6.9 ± 1.8	4.1 ± 2.1
MDA-MB-231	212.6 ± 36.6	62.6 ± 18.7	73.1 ± 2.9	40.4 ± 2.5	36.9 ± 3.1	8.8 ± 2.5	32.2 ± 9.5	5.5 ± 2.3	9.2 ± 0.5

N/D (non-defined) marks cases where IC_{50} activity falls out of tested concentration range.

** SEM was not defined due to specifics of data analysis methodology.*

Table S4. Cytotoxic activity of LMB002-closed, LMB002-open and gramicidin S monitored in three-dimensional (spheroid) assay. IC_{50} values are presented as absolute value ± SEM in µM (n=5).

	**LMB002**-closed			**LMB002**-open			**Gramicidin S**		
Cell line	10 mins	24 hrs	72 hrs	10 mins	24 hrs	72 hrs	10 mins	24 hrs	72 hrs
4T1	N/D	N/D	N/D	N/D	N/D	N/D	N/D	N/D	N/D
HEK-293	24.8 ± 7.6	49.8 ± 3.3	58.4 ± 3.5	8.5 ± 0.8	18.2 ± 3.1	20.8 ± 0.9	10.9 ± 10.9	12.9 ± 1.1	9.0 ± 1.1
HeLa	22.8 ± 4.0	27.4 ± 2.6	N/D	2.2 ± 2.0	2.8 ± 3.9	N/D	2.9 ± 1.5	2.9 ± 1.6	N/D
HepG2	35.7 ± 3.6	66.0 ± 2.3	67.3 ± 2.0	11.8 ± 0.1	20.4 ± 1.1	22.8 ± 2.8	6.7*	5.5 ± 0.8	7.2*
LLC	26.2 ± 5.4	49.4 ± 2.3	46.8 ± 3.6	7.1 ± 6.6	22.3 ± 2.6	3.5 ± 1.9	27.4 ± 5.2	6.9 ± 1.8	4.1 ± 2.1
MDA-MB-231	101.3 ± 6.3	45.2 ± 11.0	22.3 ± 4.4	37.0 ± 2.3	41.0 ± 3.9	6.9 ± 2.8	26.1 ± 5.3	5.5 ± 2.3	8.5 ± 0.5

N/D (non-defined) marks cases where IC_{50} activity falls out of tested concentration range.

** SEM was not defined due to specifics of data analysis methodology.*

Figure S1. Dose-dependent curves for gramicidin S and its photocontrolled analogues tested on 4T1 cell line in two-dimensional format. Each point represents average value (n=4) with error bars depicting SEM.

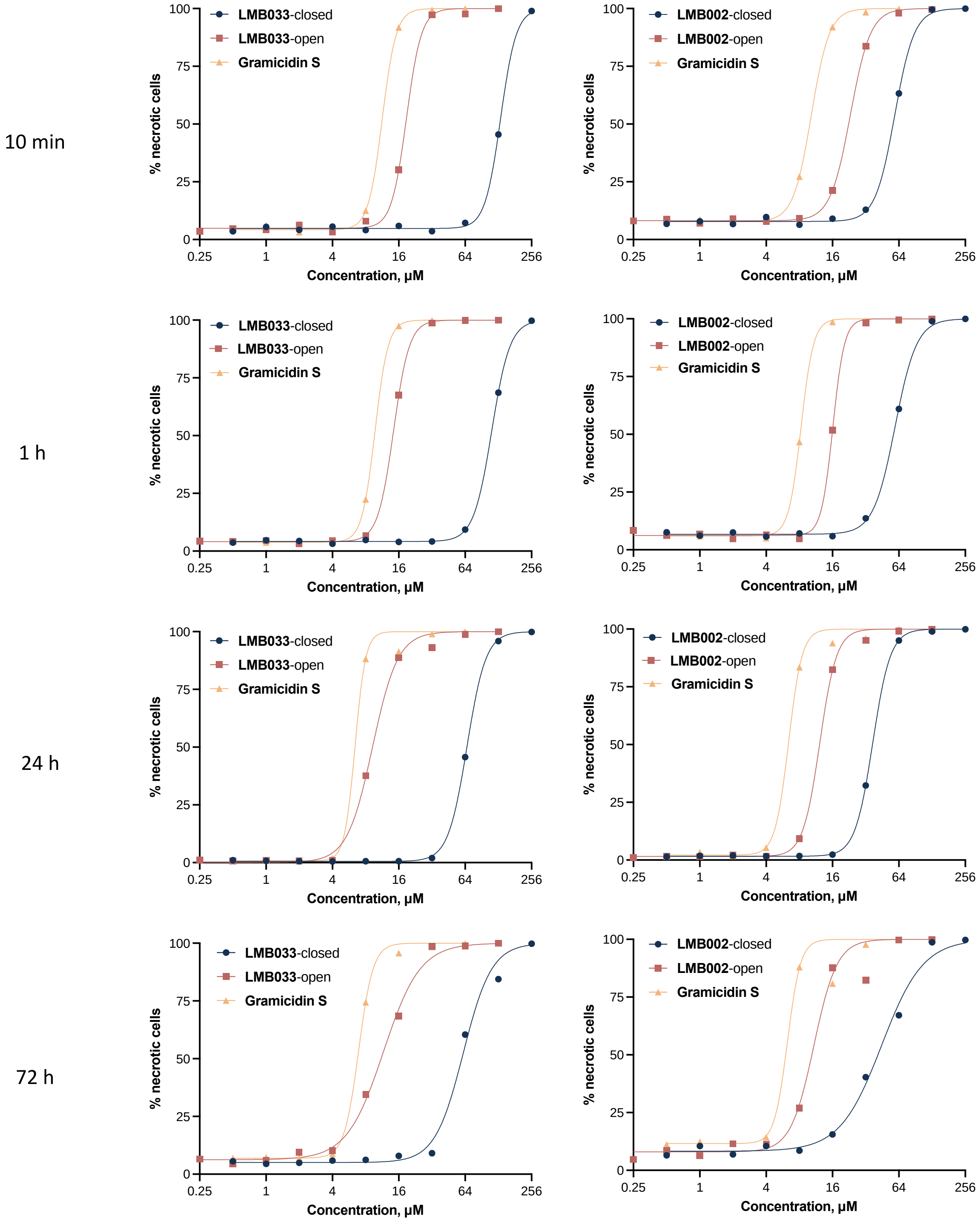

Figure S2. Dose-dependent curves for gramicidin S and its photocontrolled analogues tested on HEK-293 cell line in two-dimensional format. Each point represents average value (n=4) with error bars depicting SEM.

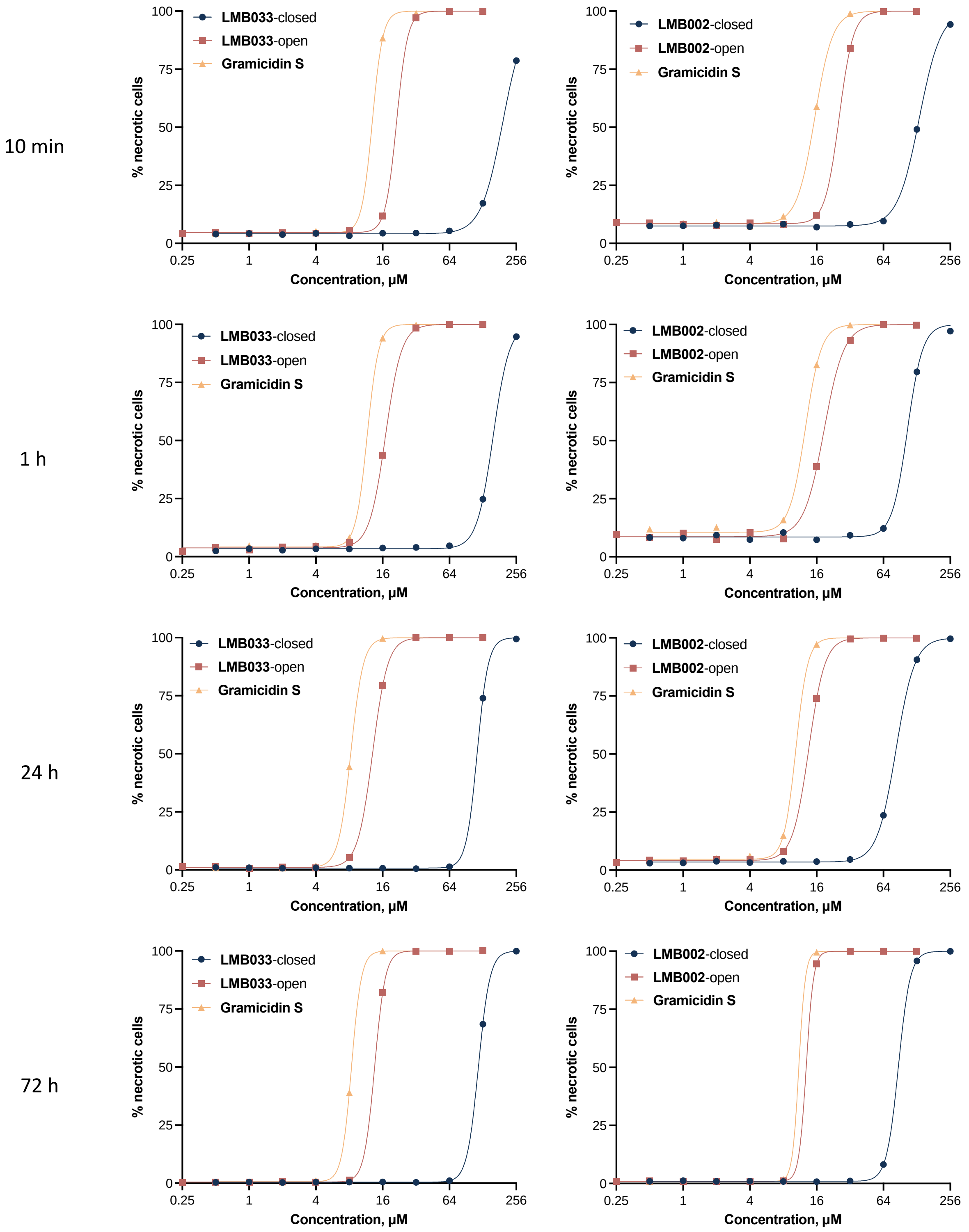

Figure S3. Dose-dependent curves for gramicidin S and its photocontrolled analogues tested on HeLa cell line in two-dimensional format. Each point represents average value (n=4) with error bars depicting SEM.

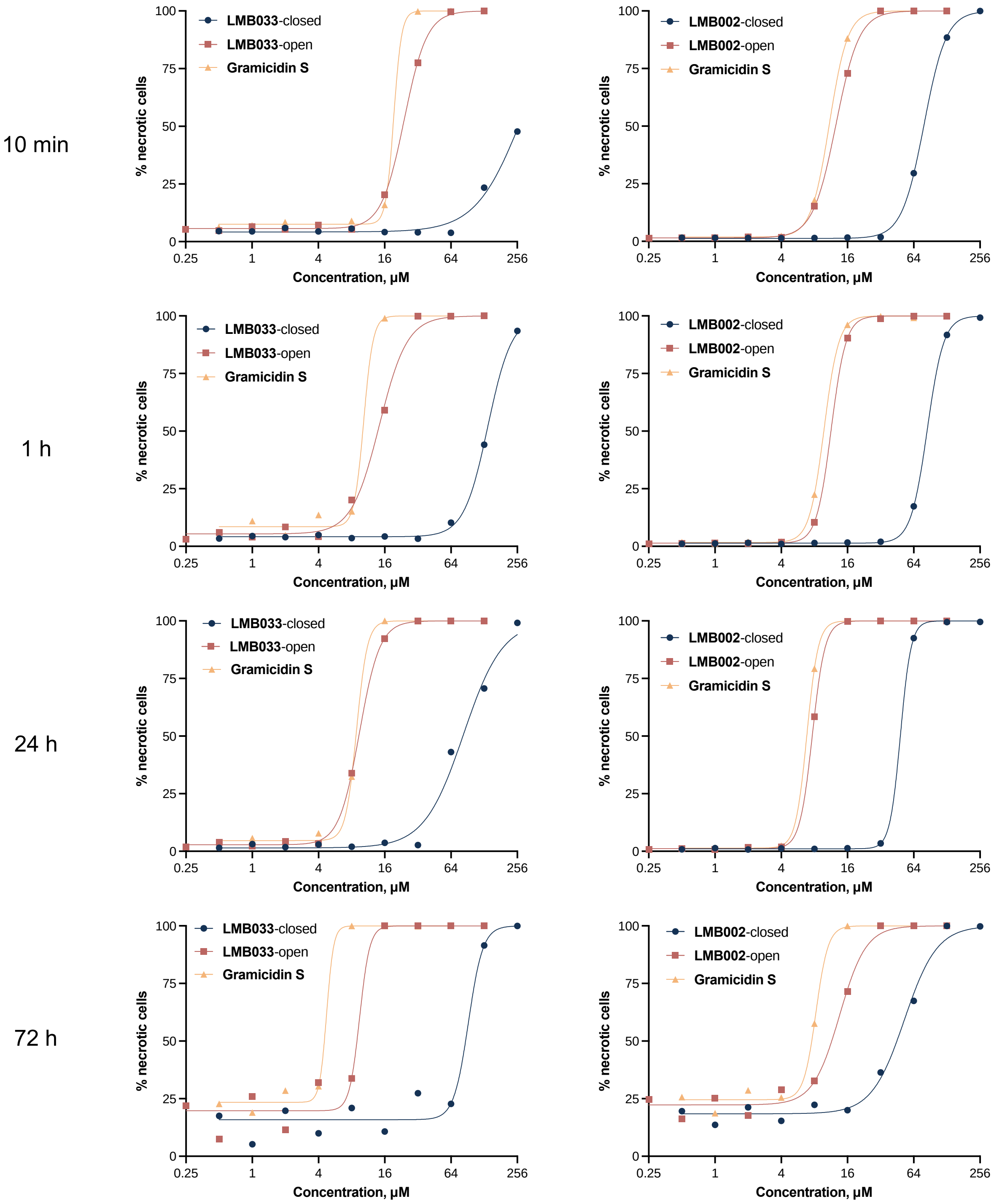

Figure S4. Dose-dependent curves for gramicidin S and its photocontrolled analogues tested on HepG2 cell line in two-dimensional format. Each point represents average value (n=4) with error bars depicting SEM.

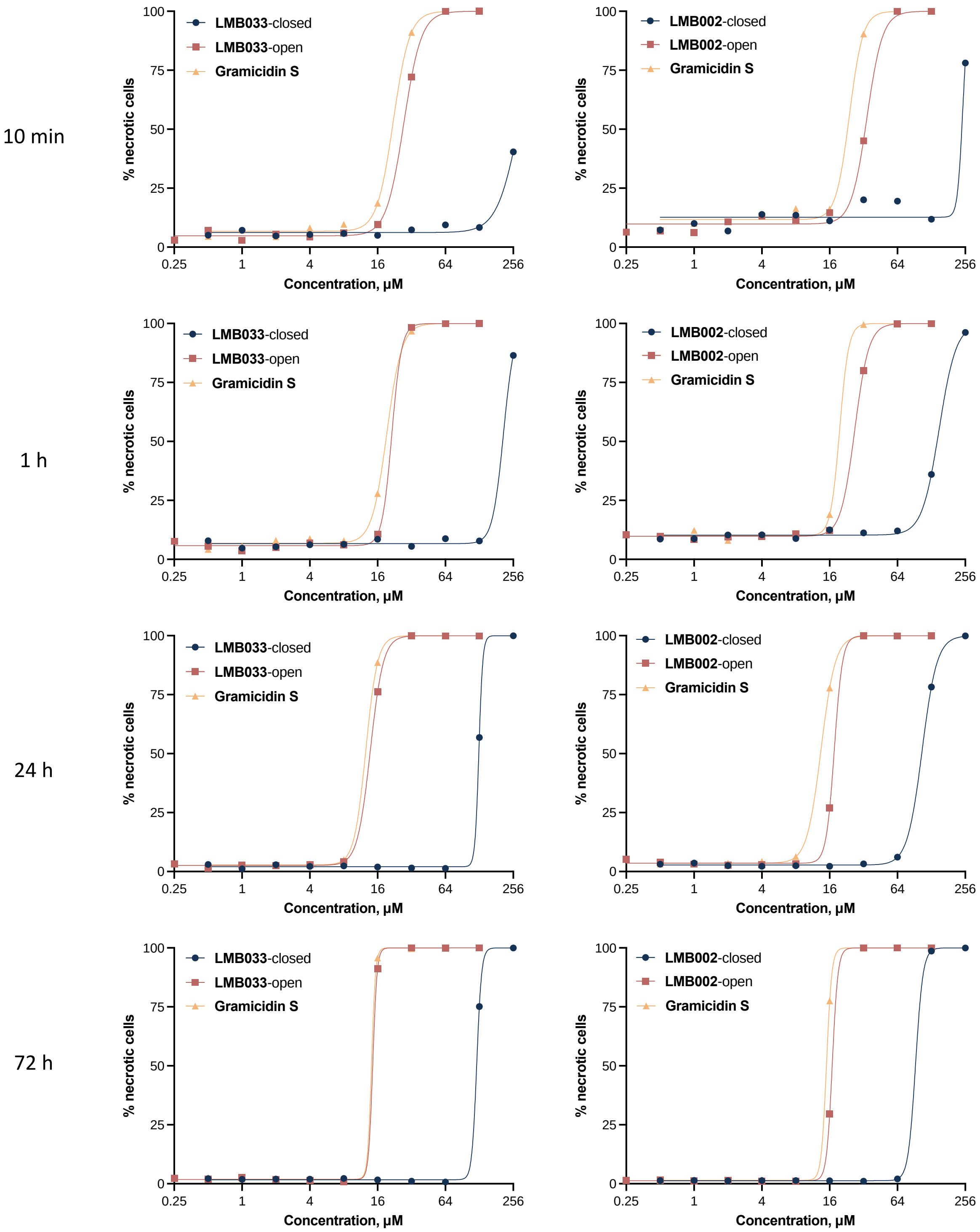

Figure S5. Dose-dependent curves for gramicidin S and its photocontrolled analogues tested on LLC cell line in two-dimensional format. Each point represents average value (n=4) with error bars depicting SEM.

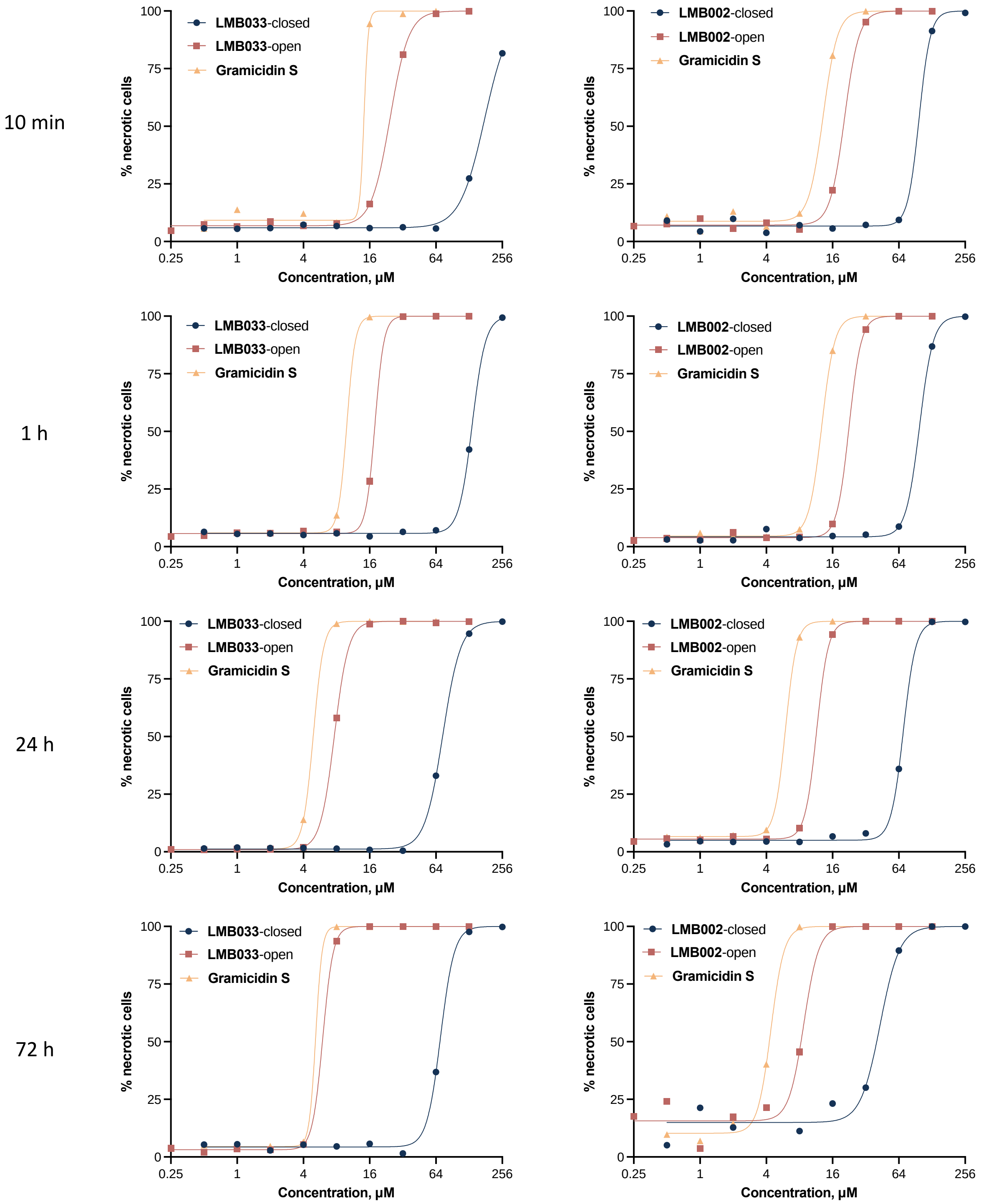

Figure S6. Dose-dependent curves for gramicidin S and its photocontrolled analogues tested on MDA-MB-231 cell line in two-dimensional format. Each point represents average value (n=4) with error bars depicting SEM.

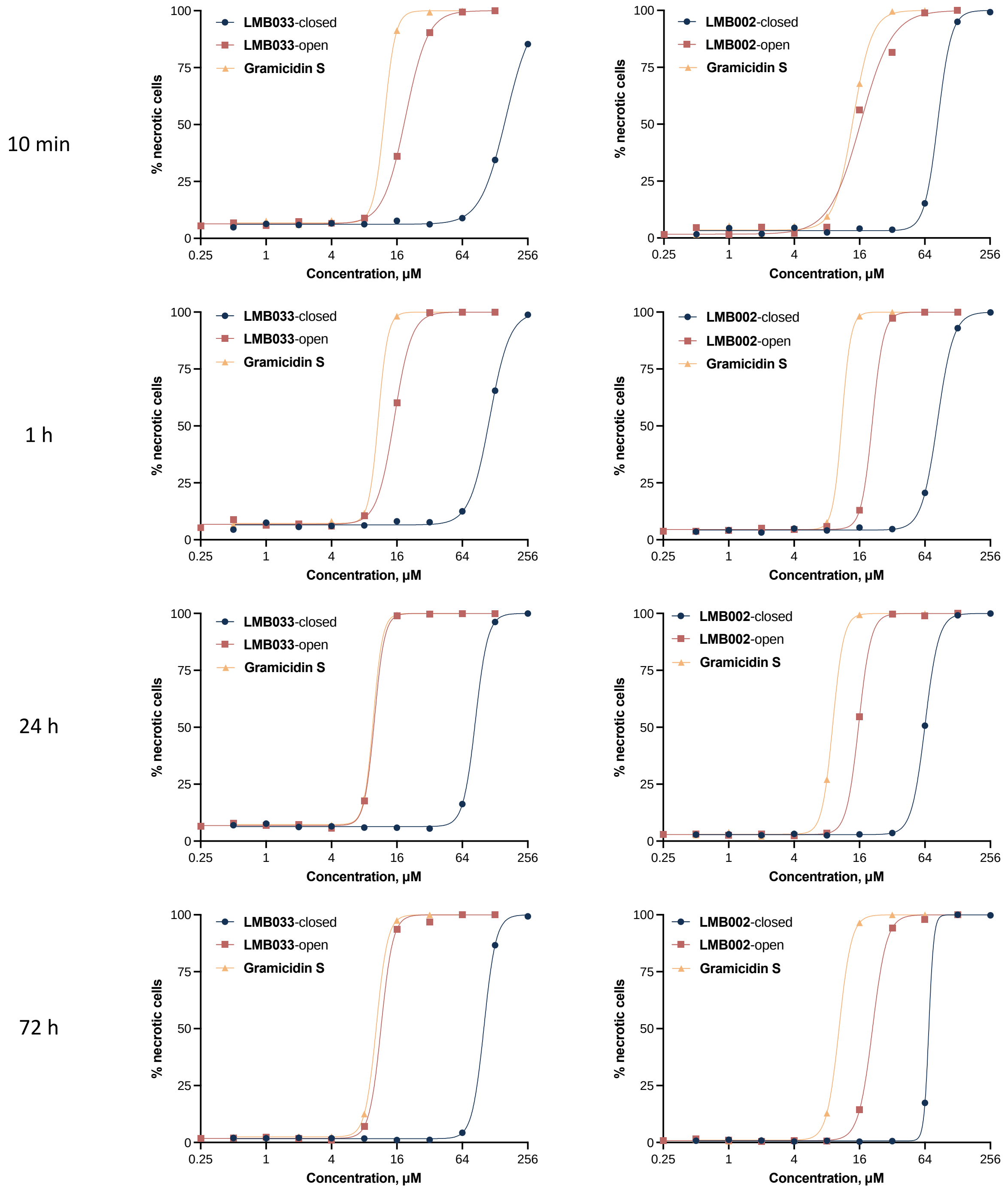

Figure S7. Dose-dependent curves for gramicidin S and its photocontrolled analogues tested on HEK-293 cell line in three-dimensional format. Each point represents average value (n=5) with error bars depicting SEM.

10 min

24 h

72 h

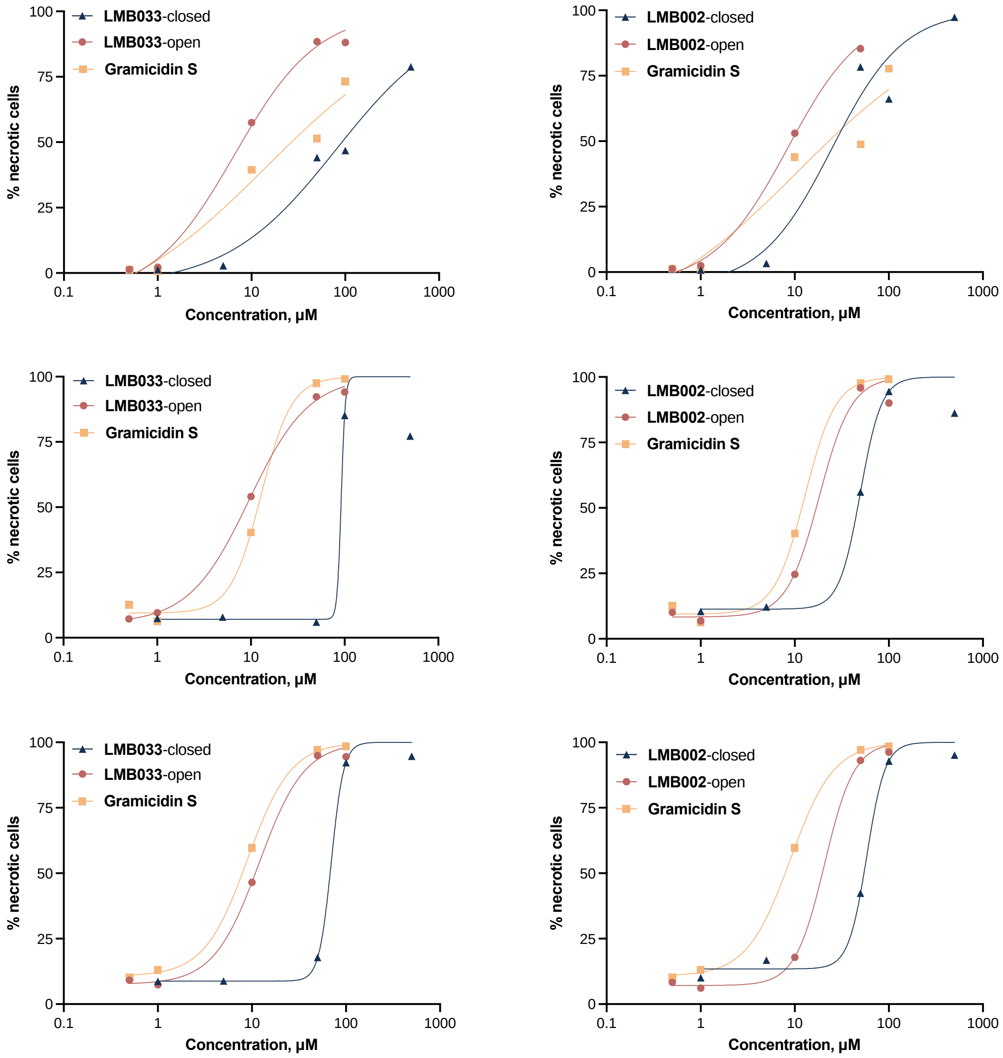

Figure S8. Dose-dependent curves for gramicidin S and its photocontrolled analogues tested on HeLa cell line in three-dimensional format. Each point represents average value (n=5) with error bars depicting SEM.

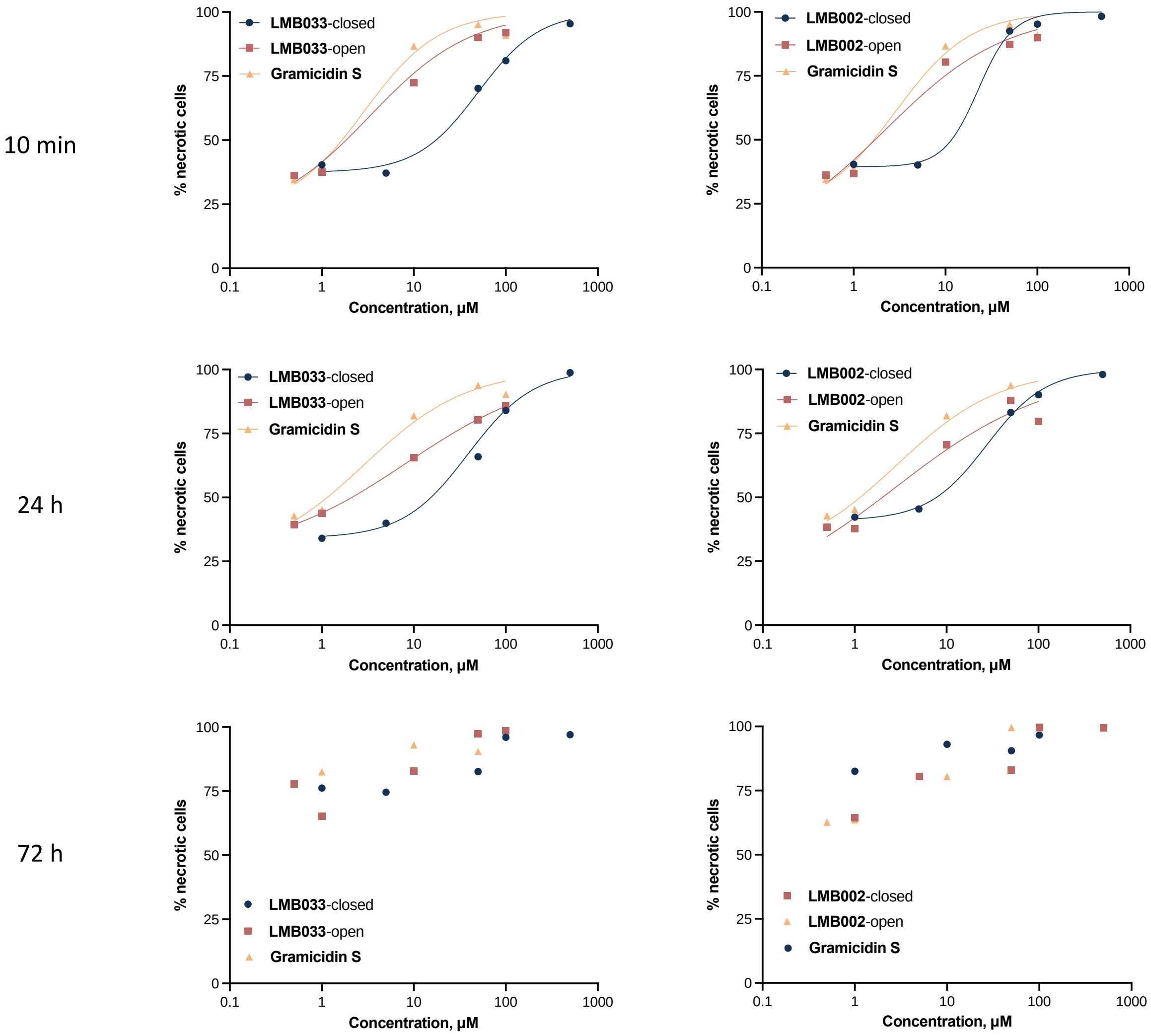

Figure S9. Dose-dependent curves for gramicidin S and its photocontrolled analogues tested on HepG2 cell line in three-dimensional format. Each point represents average value (n=5) with error bars depicting SEM.

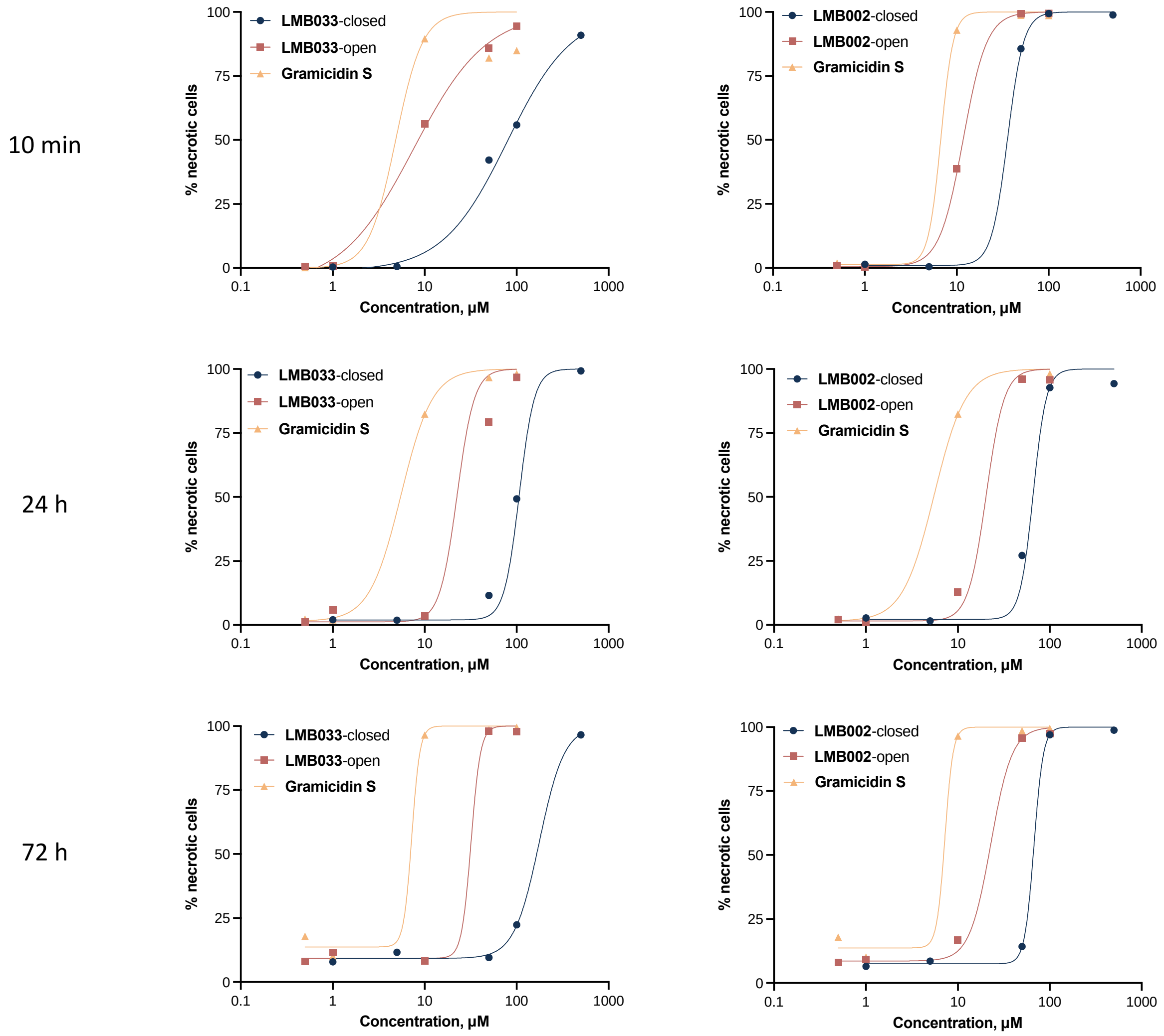

Figure S10. Dose-dependent curves for gramicidin S and its photocontrolled analogues tested on LLC cell line in three-dimensional format. Each point represents average value (n=5) with error bars depicting SEM.

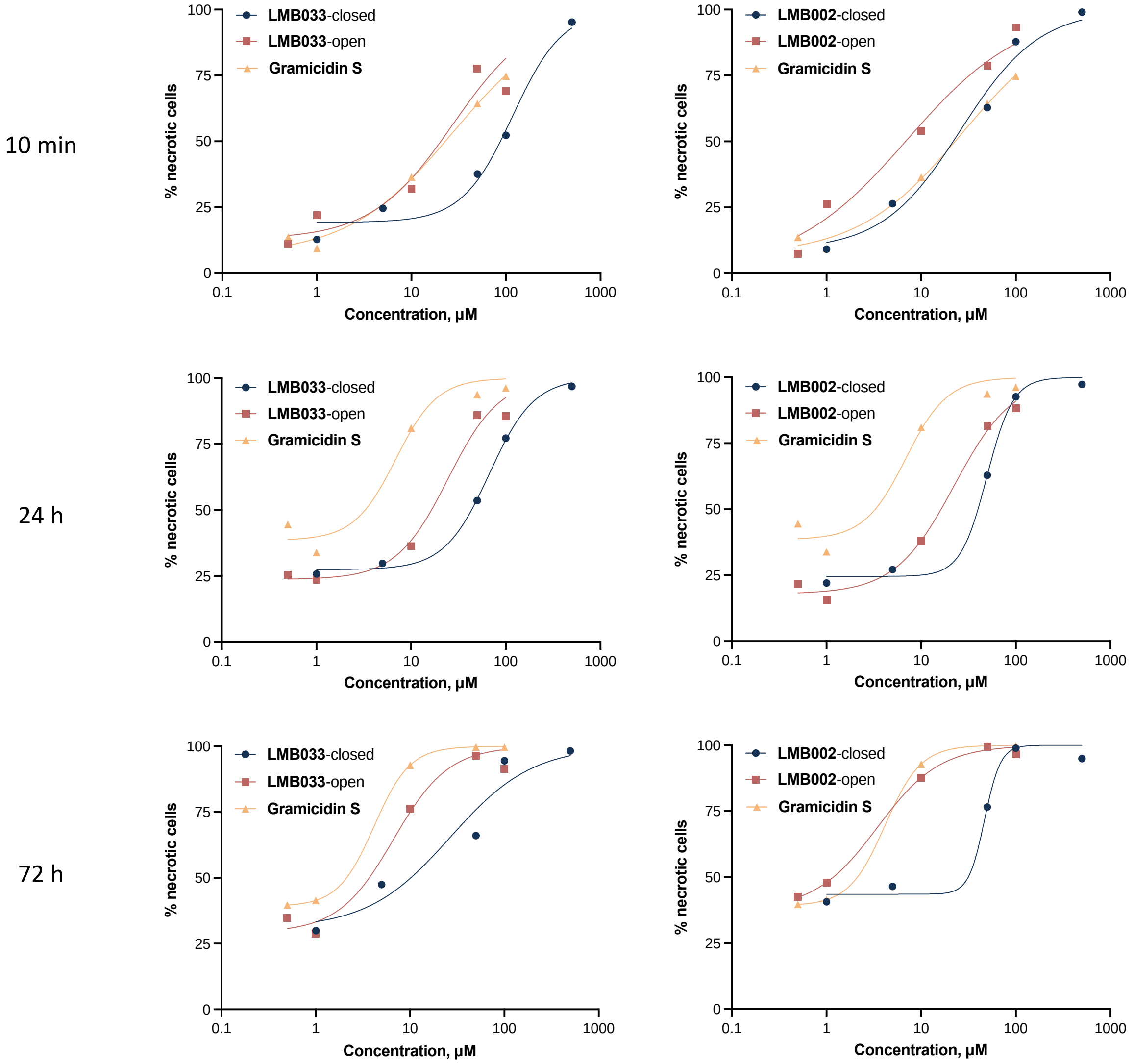

Figure S11. Dose-dependent curves for gramicidin S and its photocontrolled analogues tested on MDA-MB-231 cell line in three-dimensional format. Each point represents average value (n=5) with error bars depicting SEM.

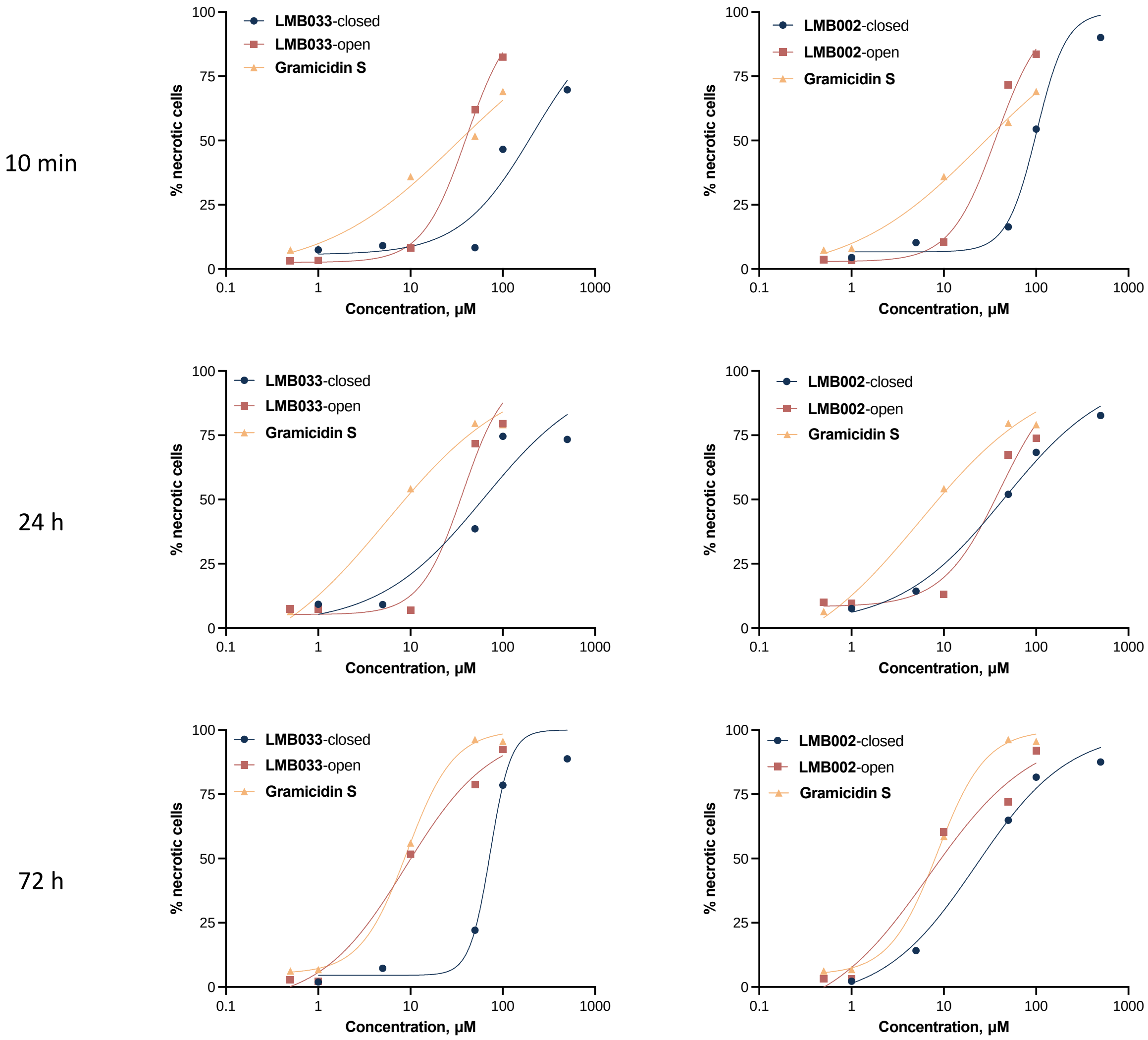

Figure S12. Kinetic curves of ATP release monitored for 1000 minutes after addition of gramicidin S and its photocontrolled analogues (run 1). Each point represents average value (n=3) with error bars depicting SEM.

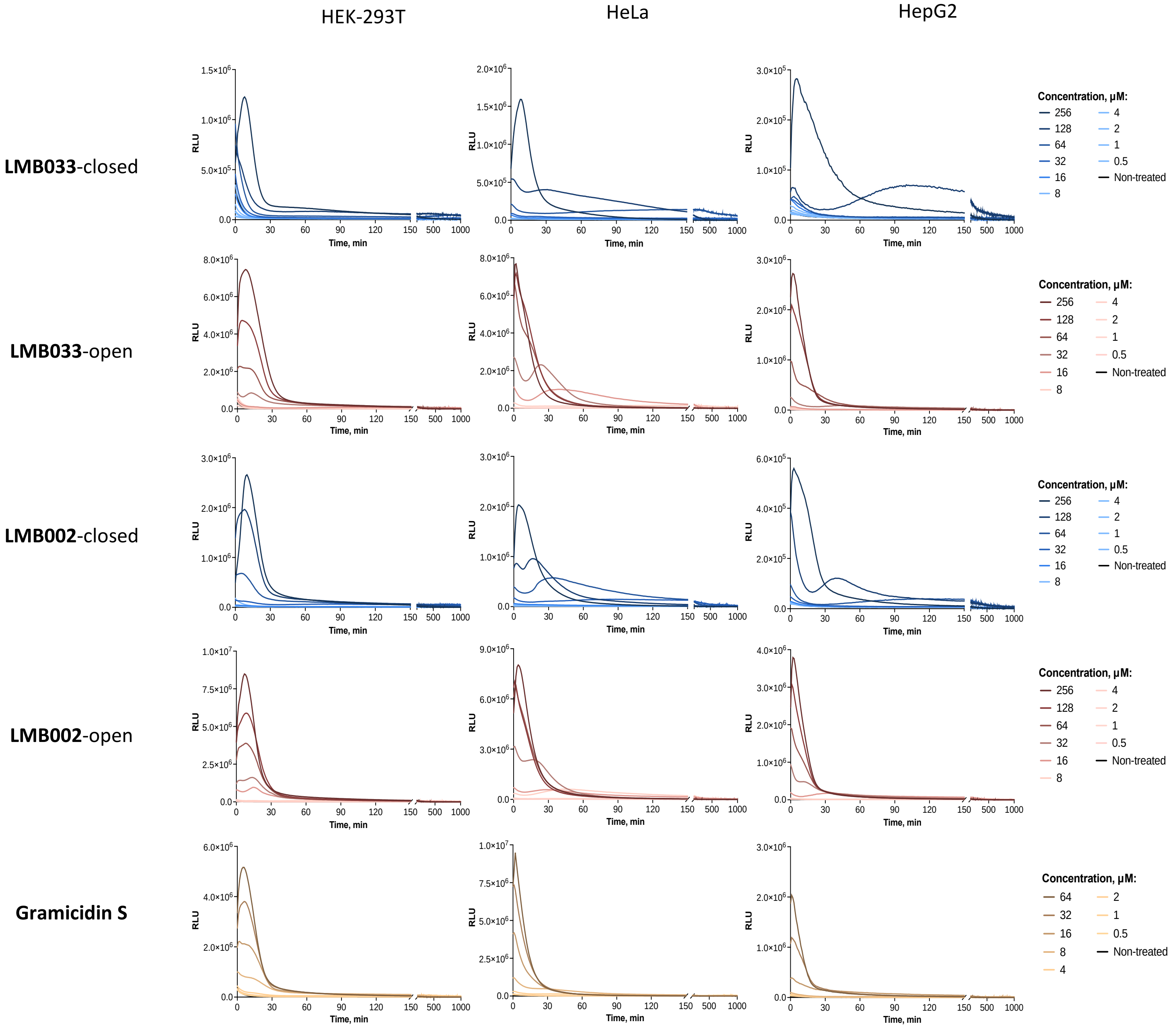

Figure S13. First derivatives of kinetic curves of ATP release monitored for 1000 minutes after addition of gramicidin S and its photocontrolled analogues (run 1). Each point represents average value (n=3) with error bars depicting SEM.

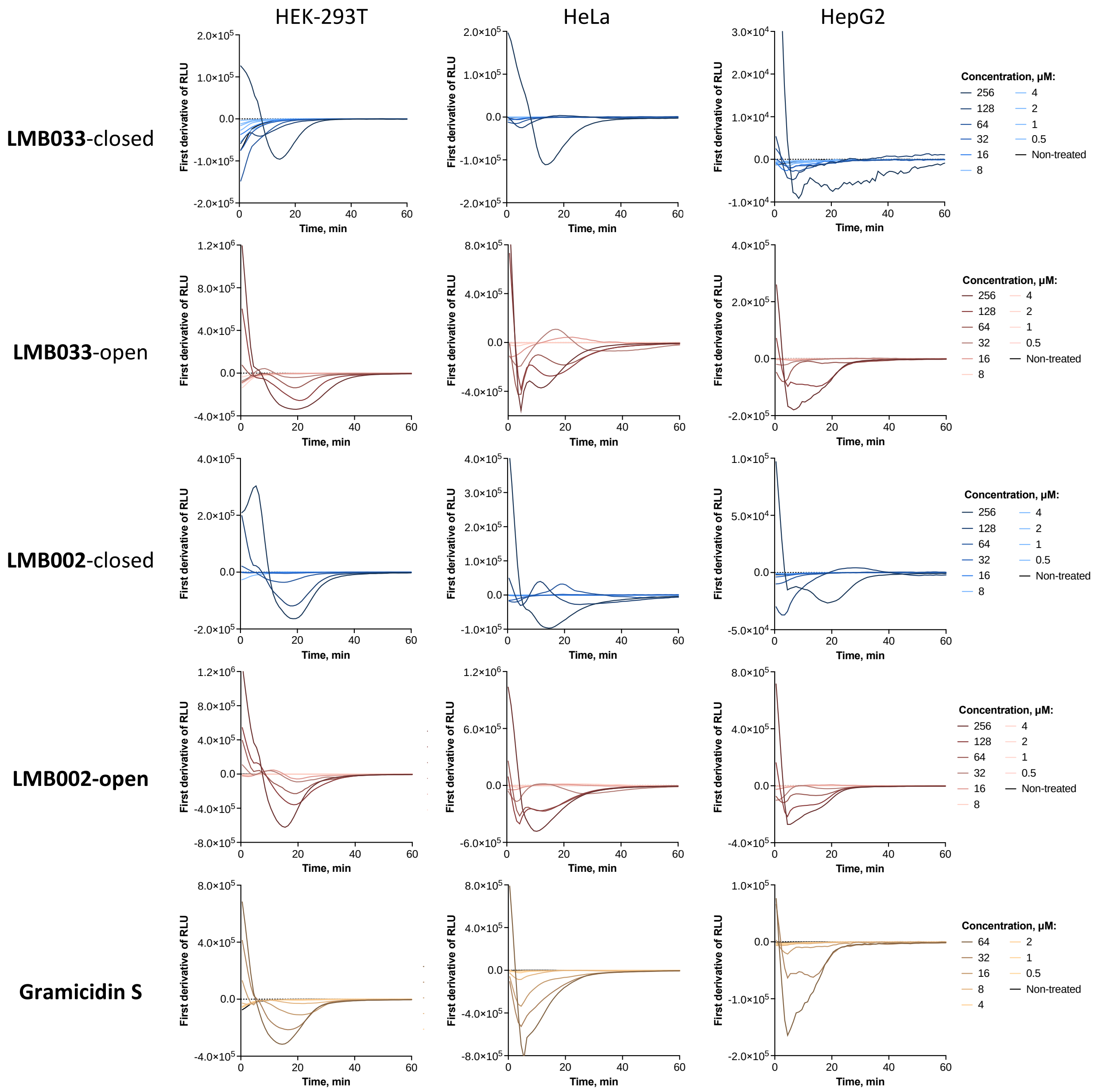

Figure S14. Kinetic curves of ATP release monitored for 1000 minutes after addition of gramicidin S and its photocontrolled analogues (run 2). Each point represents average value (n=3) with error bars depicting SEM.

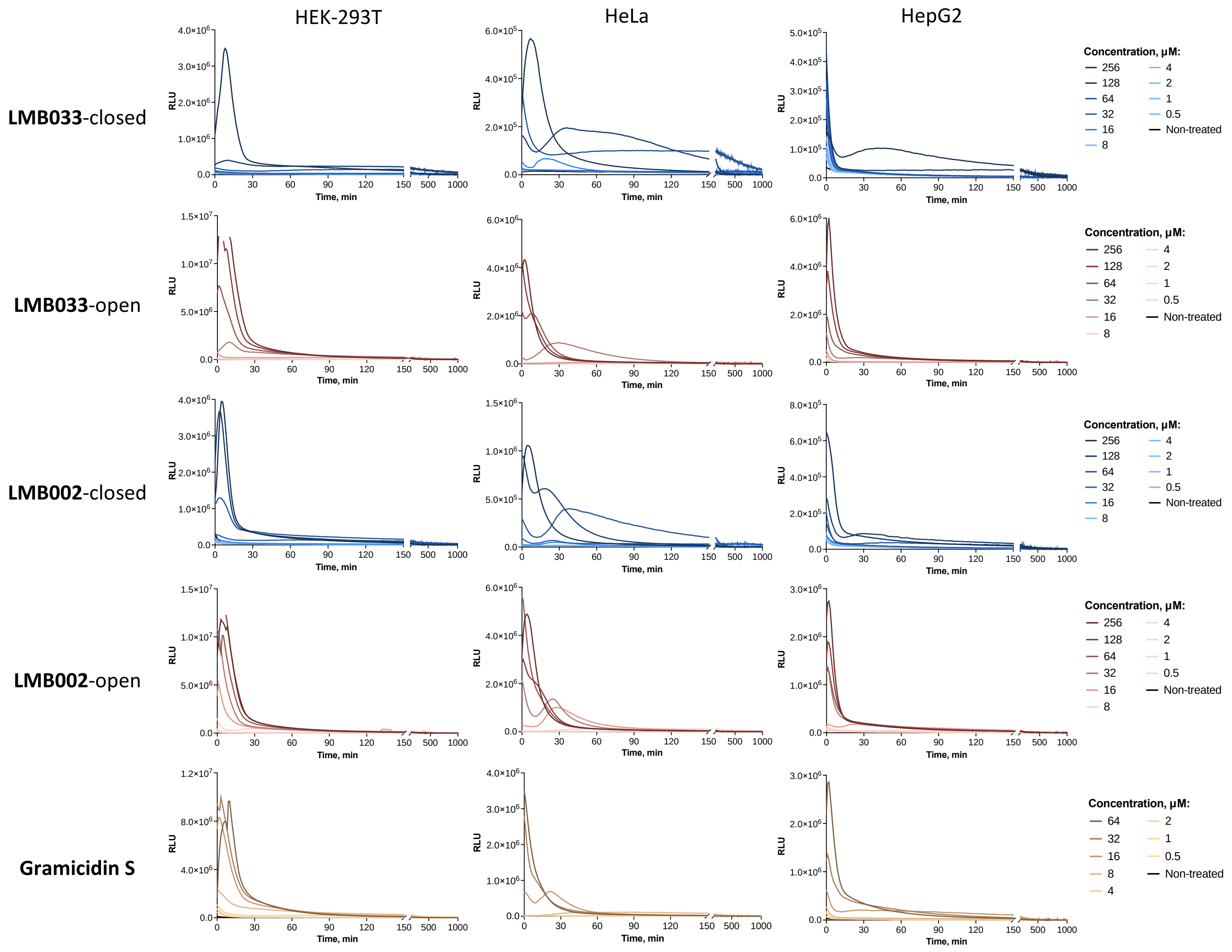

Figure S15. Area-under-curve (AUC) histrograms of ATP release monitored for 1000 minutes after addition of gramicidin S and its photocontrolled analogues (run 2). Each point represents average value (n=3) with error bars depicting SEM.

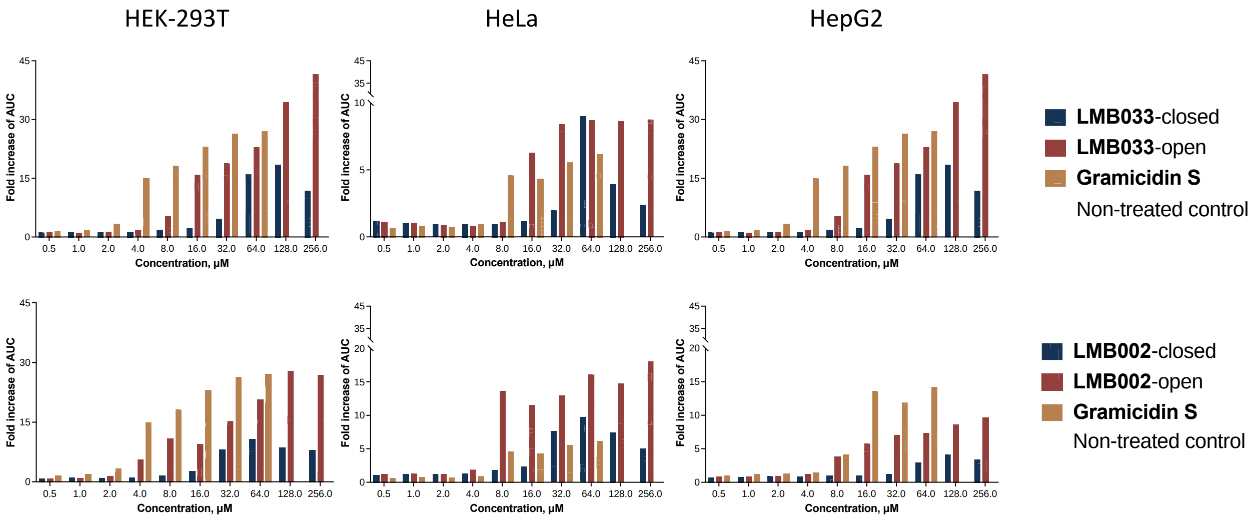

Figure S16. First derivatives of kinetic curves of ATP release monitored for 1000 minutes after addition of gramicidin S and its photocontrolled analogues (run 2). Each point represents average value (n=3) with error bars depicting SEM.

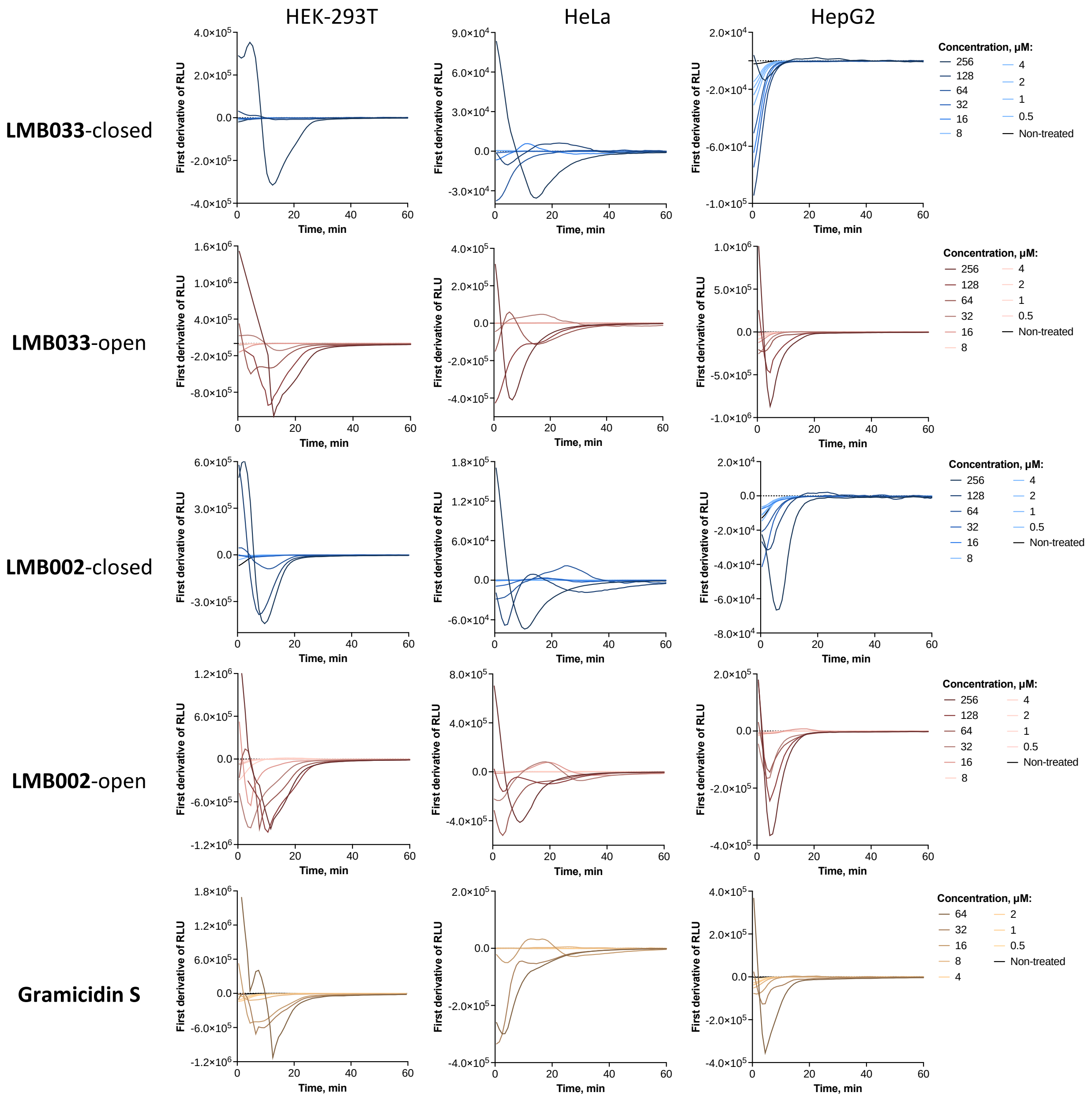

Figure S17. Scatterplots of MDA-MB-231 cells treated with LMB033-open at concentration of 4, 8, 16 and 32 µM for 30 mins, 3 hours, 6 hours and 24 hours with antibody-staining for membrane-bound CALR. The percentages on each graph illustrate fraction of cells gated as CALR-positive.

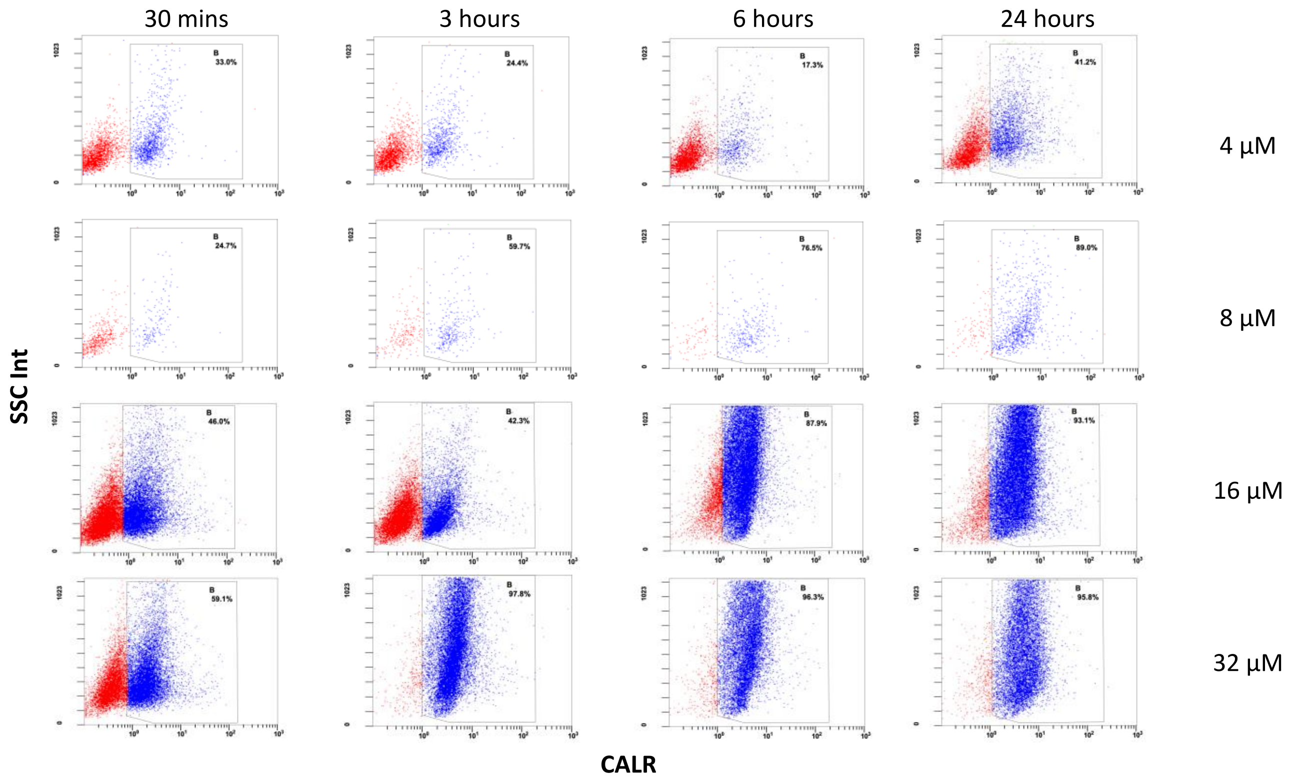

Figure S18. Scatterplots of MDA-MB-231 cells treated with LMB002-open at concentration of 4, 8, 16 and 32 µM for 30 mins, 3 hours, 6 hours and 24 hours with antibody-staining for membrane-bound CALR. The percentages on each graph illustrate fraction of cells gated as CALR-positive.

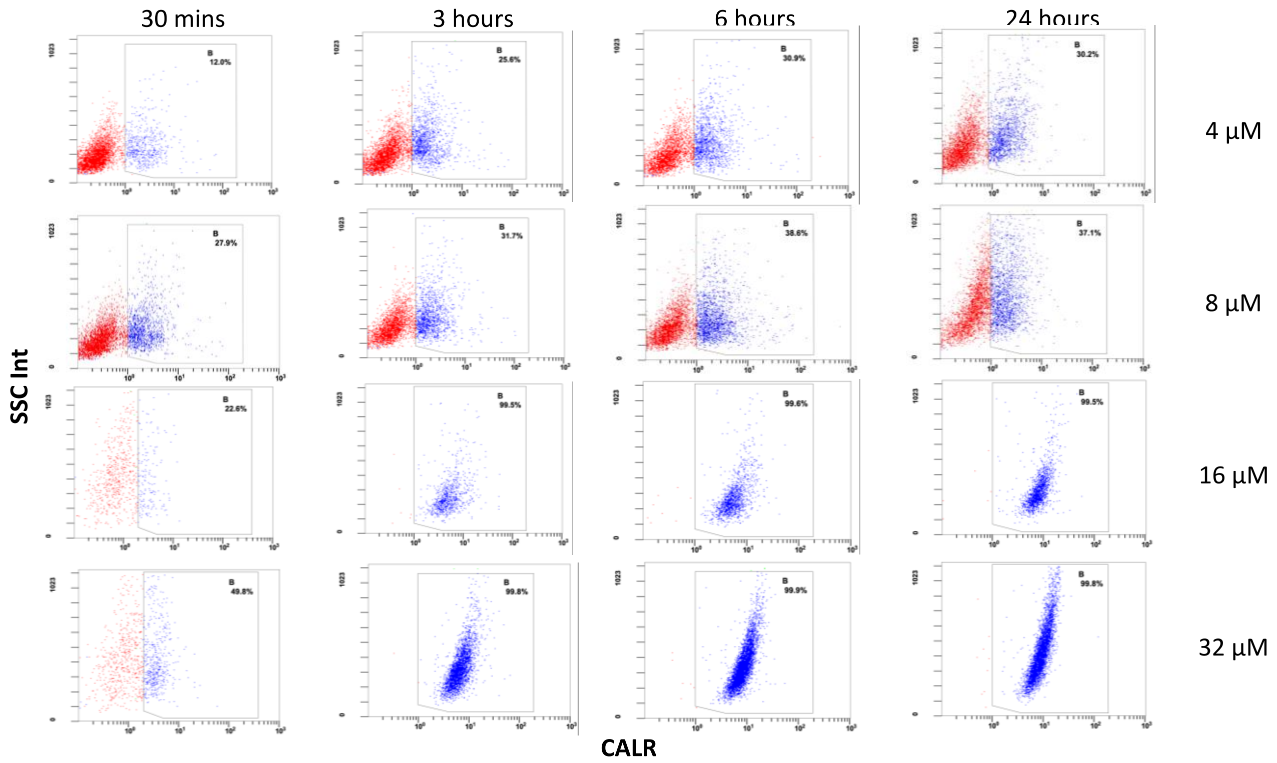

Figure S19. Scatterplots of MDA-MB-231 cells treated with gramicidin S at concentration of 4, 8, 16 and 32 µM for 30 mins, 3 hours, 6 hours and 24 hours with antibody-staining for membrane-bound CALR. The percentages on each graph illustrate fraction of cells gated as CALR-positive.

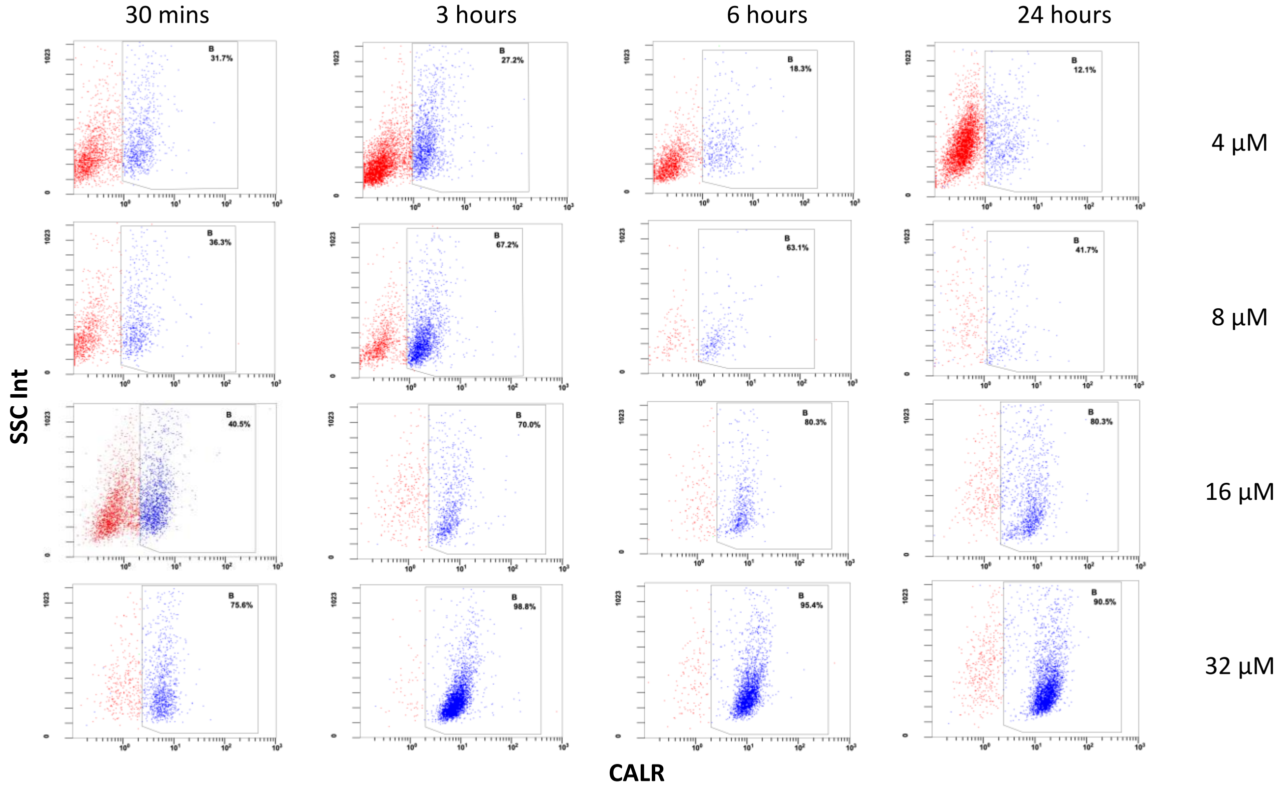

Supplement: MD-016-D5MD00075K-s001 [file MD-016-D5MD00075K-s001.pdf]
